# Structural and Functional Characterization of PA14/Flo5-Like Adhesins From *Komagataella pastoris*

**DOI:** 10.3389/fmicb.2018.02581

**Published:** 2018-10-30

**Authors:** Michael Kock, Stefan Brückner, Nina Wozniak, Manuel Maestre-Reyna, Maik Veelders, Julia Schlereth, Hans-Ulrich Mösch, Lars-Oliver Essen

**Affiliations:** ^1^Department of Biochemistry, Faculty of Chemistry, Philipps University of Marburg, Marburg, Germany; ^2^Department of Genetics, Faculty of Biology, Philipps University of Marburg, Marburg, Germany; ^3^Institute of Biological Chemistry, Academia Sinica, Taipei, Taiwan; ^4^LOEWE Center for Synthetic Microbiology, Philipps University of Marburg, Marburg, Germany

**Keywords:** adhesins, cell wall, β-*N*-acetylglucosamine capped glycans, carbohydrate-binding protein, glycan specificity, lifestyle adaptation, biotechnology

## Abstract

Cell–cell and cell-substrate based adhesion of yeasts are major determinants of their adoption of different life styles. Genome-mining of ascomycetous GPI-anchored cell wall proteins with lectin-like PA14 domains identified a unique class of putative adhesins in the clade of methylotrophic *Komagataella* yeasts, many of which are known to colonize plants and insects involving yet unknown adhesion mechanisms. Here, we report the functional and structural analysis of two of its members: *Kp*Flo1 (=Cea1), that is highly specific for terminal *N*-acetylglucosamine moieties, and *Kp*Flo2, which represents an orphan lectin with intact binding site but unknown specificity. Crystal structures of the Cea1 adhesion domain complexed to *N*-acetylglucosamine and *N*,*N*′-diacetylchitobiose reveal a Ca^2+^-dependent binding mode that differs from other members of the PA14/Flo5 adhesin family. Heterologous expression of Cea1A in *Saccharomyces cerevisiae* promotes cellular adhesion to non-reducing ends of non-crystalline chitin. Overall, our data suggest that high-affinity recognition of β-GlcNAc-capped glycans by Cea1 enable *Komagataella* species to interact with surface cues present in fungi and insects.

## Introduction

Fungi colonize most known ecological niches and hence are nearly ubiquitously found on Earth. Accordingly, fungi can develop different uni- and multicellular life forms in response to changing environmental conditions. An important factor contributing to fungal versatility is their cell wall, which is not only crucial for fungal cell–cell and cell–substrate interactions, but also for acting as physical barrier against host defense systems. The importance of fungal cell walls in establishing self-interactions and symbiosis/pathogenicity is underscored by their complex structure and composition. In *Saccharomyces* species, up to 30% of the cell’s dry weight consists of wall material composed of β1,3- and β1,6-glucans, β1,3-glucan-chitin complexes and many heavily glycosylated mannoproteins including GPI-anchored cell wall proteins (GPI-CWP) (Klis et al., 2002). In *Saccharomyces* and other yeast species, GPI-CWPs act as constitutive cell wall components or as hydrolases (Pittet and Conzelmann, 2007). Many of them are adhesins that enable self-recognition as exemplified by the well-known Flos of *Saccharomyces cerevisiae*. These Flos confer specific aggregation of vegetative cells into protective structures like flocs or biofilms as an early example of social behavior among lower eukaryotes. While Flo11-like adhesins mediate homotypic interactions by a hydrophobically decorated fibronectin type III domain (Kraushaar et al., 2015), specific ligand binding by adhesive GPI-CWPs is often mediated by the widely distributed PA14 family domain. In pathogenic yeasts of the *Candida* clade like *Candida glabrata*, GPI-CWP-type adhesins harboring PA14 domains mediate host-microbe interactions and are crucial factors for establishing pathogenicity.

A hallmark of GPI-CWP type adhesins is their modular architecture. In general, they harbor an *N*-terminal A domain that is followed by a region carrying a highly variable number of serine/threonine-rich tandem repeats (B region). The *C*-terminal domain (C domain) carries the GPI-anchor that confers covalent fixation to the cell wall by transglycosylation to β1,6-glucans. The A domain that is crucial for adhesion comprises the binding site, which, in the case of PA14 domain-related adhesins, recognizes disaccharidic ends of glycans in a C-type lectin-like manner. Binding is achieved via a Ca^2+^-ion being complexed by a unique D*cis*D motif and further amino acid residues in two calcium binding loops (CBL1, CBL2). For example, the Flo-5 A domain of *S. cerevisiae* (*Sc*Flo5A) mediates specific adhesion to Man-α1,2-Man moieties presented on the surface of other yeast cells and thereby causes floc formation by cell–cell interactions (Veelders et al., 2010). A second, homophilic mode of A domain interaction has been described for *Sc*Flo1A that resembles Flo5 in term of its specificity for terminal mannosyl residues. Here, direct calcium-bridging between A-domain derived glycans could be additionally observed, which may represent an early, less specific step during formation of multicellular aggregates (Goossens et al., 2015). In the case of PA14/Flo5-like adhesins of the large Epa family from *C. glabrata*, the A domains recognize terminal galactosides in the glycocalyx of epithelial cells, leading to pathogenic host-cell adhesion (Maestre-Reyna et al., 2012; Diderrich et al., 2015).

Five different 3D structures of PA14/Flo5-like adhesin domains have been reported including *Sc*Flo5A (PDB-ID 2XJP), Lg-Flo1A (4GQ7), *Sc*Flo1A (4LHN), Epa1A (4ASL), and Epa6A (4COU). Despite revealing the common PA14-like β-sandwich and D*cis*D motif these structures showed different modes of carbohydrate recognition (Petosa et al., 1997; Veelders et al., 2010; Ielasi et al., 2012; Maestre-Reyna et al., 2012; Sim et al., 2013; Goossens et al., 2015). These adhesins confer either self-recognition (Flo) or host-recognition (Epa), and can be divided into two different structural subgroups. Interestingly, genes encoding Flo or Epa adhesins are found to undergo intense intergenic recombination, thereby promoting the generation of chimeric adhesins and a high degree of functional variability (Christiaens et al., 2012).

In this study, we mined the genomes of ascomycetous fungi for hitherto unknown PA14/Flo5-like adhesin domains and identified such domains in several putative GPI-CWP adhesins of methylotrophic yeasts from the *Komagataella* genus. Despite their biotechnological application for heterologous protein production, the natural lifestyles and adhesion properties of *Komagataella* species like *Komagataella pastoris* and *K. phaffii* are largely unknown (Ogata et al., 1969; Mbawala et al., 1990; Daly and Hearn, 2005). Compared to other recombinant expression systems, both species, initially assigned as different *Pichia pastoris* strains, are distinguished by their high secretion capacity, the availability of strains with humanized glycosylation patterns and the dependence on simple carbon sources such as methanol for achieving high biomass yields. *K. pastoris* has first been isolated from decomposing wood; other yeasts of *Komagataella* clade have been identified as part of the gut flora of insects (Shihata and Mrak, 1952; Phaff and Knapp, 1956; Kurtzman, 2011). Here, we performed a detailed structural and functional characterization of A domains from putative GPI-CWP adhesins of the *K. pastoris* reference strain DSMZ70382, previously known as *P. pastoris*. Our data show that the A domain of one of these GPI-CWP, chitin-end adhesin 1 (Cea1), binds to *N*-acetylglucosamine or at non-reducing ends of chitinous polymers with high affinity *in vitro* and *in vivo*. Furthermore, *K. pastoris* A domains share features from both previously characterized Flo and Epa subgroups. Among them Cea1A represents a novel subgroup of adhesive PA14 domains, which mediate high affinity recognition of chitinous poly- and oligomers.

## Experimental Procedures

### Phylogenetic Analysis of PA14-Domain Containing Putative Adhesins of *K. pastoris*

**FIGURE 1:**
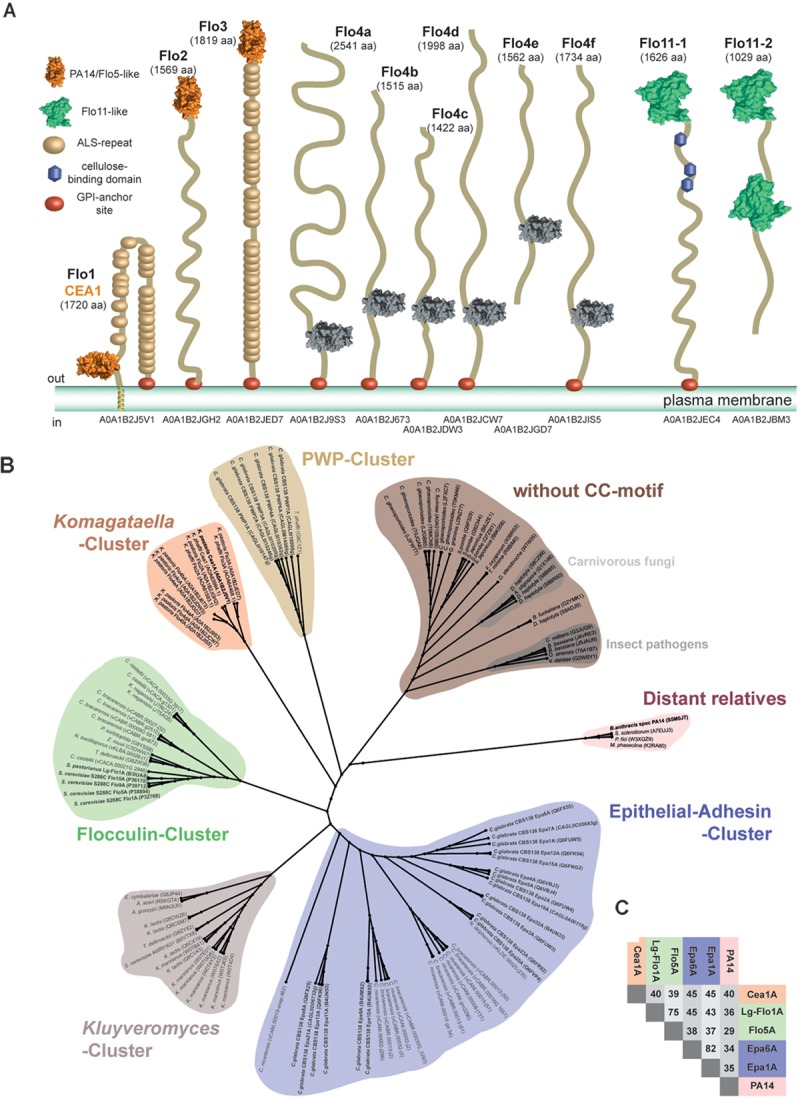
Phylogenetic analysis of ascomycetous PA14/Flo5-like GPI-CWP adhesins. **(A)** Domain structures of PA14- and Flo11-type cell wall proteins of *Komagataella pastoris* as found in the revised genome of strain DSMZ 70382. Notably, the PA14_2 domain of Flo4a-Flo4f lacks any disulfide links like the CC-motif devoid subgroup, but they are clearly higher related to other *Komagataella* PA14-like adhesin domains. **(B)** The A domains of PA14 domain-containing fungal adhesins of *Saccharomyces cerevisiae* and *Candida glabrata* were compared to newly identified putative adhesins of *K. pastoris* and further PA14_2 domain containing putative adhesins from the Pfam database by constructing a phylogenetic ML tree on the basis of a T-Coffee alignment. The PA14 domain of the *Bacillus anthracis* protective antigen was used as outlier in the phylogenetic analysis and clusters together with further distant relatives (red). A small, hitherto unknown subgroup of PA14/Flo5-like A domains is formed by gene products from *Komagataella* (*Komagataella*-cluster, orange). The flocculins of *S. cerevisiae* cluster in a small subgroup (green); epithelial adhesins (EPA) in another, more diverse subgroup (blue). Smaller clusters are found for PWPs from *C. glabrata* (yellow) and putative adhesins mainly from *Kluyveromyces* species (gray). A separate, large group of PA14/Flo5-like proteins lacks a conserved motif of two consecutive cysteine residues present in the other clusters (brown). Putative GPI-CWP adhesins from *K. pastoris* strain DSMZ70382 are shown according to their ORF number (www.pichiagenome.org), and sequences from diverse *Candida* orthologs are indicated according to Gabaldon et al. (2013). Entries of characterized gene products are shown in bold letters. **(C)** Sequence identities of selected members of the three fungal PA14/Flo5-like adhesin subgroups: Flocculin-cluster (green), EPA-cluster (blue), *Komagataella*-cluster (orange), and the PA14-domain from *B. anthracis* (red).

To identify further members of the fungal PA14/Flo5-like GPI-CWP adhesin superfamily, an approach combining database search with genome mining was used. A domain sequences of *Sc*Flo5 (Uniprot: P38894) and Epa1 (Uniprot: Q6VBJ0) were used as seeds to identify further non-annotated homologs using BLAST tools on ascomycetous genomes (tax id: 4890). Based on the revised genome data of *Komagataella* species by Love et al. (2016) we identified nine PA14-domain containing GPI-CWPs in *K. pastoris* and seven in the related *K. phaffii* strain. In a second step designated A domains sequences were aligned by T-Coffee (Notredame et al., 2000) by including orthologous Flos from *S. cerevisiae* S288c, Epa-adhesins and Pwp A domains (Desai et al., 2011) from *C. glabrata* CBS138, a subgroup of putative adhesins from *Candida* species (Gabaldon et al., 2013) and the PA14 domain from the *Bacillus anthracis* protective antigen (Petosa et al., 1997). After alignment of these PA14/Flo5-like GPI-CWP adhesins the MAFFT-add algorithm was applied to all sequences of putative A domains from the Pfam family PA14_2 (=GLEYA domain; Pfam-ID: PF10528), which were filtered before for sequences containing only one terminal orthologous domain. After deletion of misaligned sequences the remaining sequences were realigned. A maximum likelihood phylogenetic tree was constructed using MEGA6 (LG-model with gamma distribution, partial deletion of gaps, nearest-neighbor-interchange) (Tamura et al., 2013); the tree was visualized with FigTree 1.4^1^ (Figure 1B).

### Cloning, Overexpression and Purification of *K. pastoris* a Domains

In *K. pastoris* strain DSMZ 70382 the Cea1A (=*Kp*Flo1A) domain belongs to a predicted adhesin with PA14 domain (D78-E296, Uniprot: A0A1B2J5V1); likewise the *Kp*Flo2A domain resides at the N-terminus of another GPI-CWP (Q33-A253, Uniprot: A0A1B2JGH2). Gene fragments coding for these A domains were amplified from genomic DNA using primers 5′-CAGTCGACATATGGATGACAGTGGAAATGG/5′-GCTCGAGTTATTCATGGCAGGAGTTCTC for Cea1A and 5′-CAAGTTACATATGCAGGAAAGTGGTGATGG/5′-GCTCGAGTTATGCTTGGCATTGTTCTTC (Metabion) for *Kp*Flo2A, respectively. These fragment were subcloned into pET28a (Novagen) using *Nde*I and *Xho*I restriction sites (underlined in primer sequence) to encode *N*-terminally His_6_-tagged A domain fusions. Heterologous overexpression was performed according to Veelders et al. (2010) in *Escherichia coli* SHuffle T7 Express (New England Biolabs). Cells were grown at 37°C in TB medium to an OD_600_ = 0.2 and were subsequently cooled to 12°C. Overexpression was induced by addition of 10 μM IPTG when the culture reached OD_600_ = 0.6. After harvesting, cell pellets were resuspended in AM-buffer (100 mM Tris-HCl, 200 mM NaCl, pH 8.0), frozen in liquid N_2_ and stored at -80°C. After thawing and addition of lysozyme, PMSF, EDTA and DNAseI cell disruption was performed using a French press (Aminco). After centrifugation the supernatant was applied to a Ni-NTA column (Macherey Nagel); the recombinant *Kp*Flo A domains were eluted with AM-buffer containing 150 mM imidazole. Eluate fractions were checked by 12% SDS-PAGE and fractions accordingly pooled and concentrated using a 10 kDa cut-off Amicon Ultra concentrator (Millipore). Finally, size exclusion chromatography with a Superdex 200 column (GE Healthcare Life Sciences) and SEC-buffer (20 mM Tris-HCl, 200 mM NaCl, pH 8) was used to yield pure monomeric *Kp*Flo A domains. Protein solutions concentrated to 10 mg/ml (Amicon Ultra concentrator, 10 kDa cut-off) were stored at 4°C.

### High-Throughput Glycan Binding Assays

Recombinant *Kp*Flo1A (=Cea1A) and *Kp*Flo2A were fluorescently labeled using an Alexa Fluor 488 THF kit (Invitrogen) and applied to CFG array V5.1 chips at protein concentrations of 20 μg/ml and 200 μg/ml, respectively. Chip surfaces were repeatedly washed and remaining fluorescence was measured and quantified. Data and the exact procedure can be found under cfg_rRequest #2555 at the webpage of the Consortium for Functional Glycomics^2^. The pie charts for the Cea1A domain were generated using the quantity of carbohydrates belonging to a specific group. All ligands showing ≥4% relative fluorescence units (RFU) of the best binder (GlcNAc directly linked to a spacer) were used. Given that we found no binder for *Kp*Flo2A, we tested a few sugars (50 mM Glc, Gal, Lac, Man, GlcNAc) by fluorescence spectroscopy using W119 near the putative glycan binding site (distance to calcium ion: 7.3 Å) as reporter, but failed likewise to identify any significant fluorescence quench indicating that none of these carbohydrates bound (data not shown).

### Structure Determination of *Kp*Flo a Domains

Crystallization was done with a Cartesian robotic system (Genomic Solutions) using commercially available screens (Qiagen) in sitting drops containing 300 nl protein solution with 300 nl reservoir solution at either 4°C or 18°C. First Cea1A crystals grown in Li_2_SO_4_ or NaCl/PEG 8000 containing conditions lacked any diffraction. After identification of GlcNAc as cognate ligand co-crystallization was performed using 5 mM GlcNAc or 5 mM *N,N*′-diacetyl-chitobiose and 5 mM CaCl_2_ in the protein solution. Orthorhombic crystals were obtained in conditions containing MgCl_2_ and 15–30 mg/ml Cea1A. Optimization was done using the hanging drop vapor diffusion method in 24-well format and streak seeding. After 3–5 days crystals for data collection were obtained in conditions containing 100 mM MgCl_2_, 100 mM NaCl, 100 mM sodium citrate pH 3.5 and 12% PEG 4000 at 4°C. Well diffracting monoclinic crystals of the *Kp*Flo2A domain were obtained in 30 mg/ml protein, 20 mM MgCl_2_, 200 mM sodium cacodylate and 50% PEG200 at 18°C.

Crystals were picked with a Micromount (MiTeGen), soaked in mother liquor containing 15–20% glycerol as cryoprotectant and frozen in liquid nitrogen before X-ray datasets were recorded at 100 K. The Cea1A structures were solved by molecular replacement using PHASER (McCoy et al., 2007) and a trimmed homology model of Cea1A that was generated by Modeller 9v7 (Eswar et al., 2006) using Epa1A (PDB ID 4AF9) as a template. The *Kp*Flo2A structure was likewise solved using instead the Cea1A domain as template. Data processing was performed with XDS, XSCALE, PHENIX, and CCP4 (Bailey, 1994; Adams et al., 2010; Kabsch, 2010). The Cea1A structures were refined with alternating rounds of REFMAC (Murshudov et al., 1997) and Coot (Emsley and Cowtan, 2004) and NCS restraints till no further improvement of *R*_free_ was possible and no unexplained difference electron density was left for interpretation (For statistics see Table 1). The Cea1A molecule pair A/B is better defined than the C/D pair in the asymmetric symmetry unit (*B*_ave_: 15.3 vs. 19.4 Å^2^) due to comparably poorer electron density in the latter for the N-terminus, A89-V99, that takes part in molecules A/B in formation of the neck region, and the stretch K205-A217. Accordingly, the neck region defined for molecules A/B is partly missing in C/D because of a lack of the disulphide linkage between C207 and C294. The latter cysteine belongs to a C-terminal stretch that is not defined in molecules C/D (D290-E296) by electron density. Similarly, the *Kp*Flo2A structure was refined with phenix.refine (Adams et al., 2010). Figures of protein structures were generated using the molecular graphics program PyMOL v1.4.1 (DeLano, 2002) and UCSF Chimera v1.8.1 (Pettersen et al., 2004).

**Table 1:** Crystallographic statistics of Cea1A (=*Kp*Flo1) and *Kp*Flo2.

Data collection/processing	Cea1A•GlcNAc	Cea1A•*N*,*N*′-diacetylchitobiose	*Kp*Flo•2glycerol
PDB accession code	5A3L	5A3M	6HOS
X-ray source	BL14.1	BL14.3	BL14.1
	BESSY II, Berlin, Germany		
Detector	MARmosaic 225 mm		
Wavelength (Å)	0.91841	0.89120	0.91841
Space group	*P*2_1_2_1_2_1_	*P*2_1_2_1_2_1_	*C*121
Cell dimensions (*a, b, c* Å)	102.34, 106.21, 107.60	101.70, 105.35, 106.47	79.94, 103.19, 72.10, β = 113.51°
Resolution (Å)	19.84–1.66 (1.75–1.66)	19.98–1.75 (1.84–1.75)	40.67–2.15 (2.27–2.15)
Total reflections	797571	423782	107631
Multiplicity	5.8 (5.8)	4.0 (4.0)	3.7 (3.7)
Unique reflections	137801	106663	29204
*R*_merge_ (%)	9.9 (59.0)	9.9 (52.3)	7.1 (56.1)
Completeness (%)	99.6 (100.0)	92.7 (95.3)	100.0 (100.0)
*I*/σ(*I*)	14.9 (3.3)	12.4 (2.8)	11.7 (2.4)
Mosaicity (°)	0.12	0.09	0.19
Wilson B-factor (Å^2^)	10.7	8.9	32.6
**Refinement statistics**			
Resolution (Å)	19.84–1.66	19.99–1.75	39.41–2.15
*R*_factor_, *R*_free_ (%)	17.07, 20.21	18.26, 21.14	17.24, 19.89
Reflections (working, test set)	135716, 2084	105044, 1619	28177, 1005
Completeness for range (%)	99.46	92.20	99.9
Total number of atoms	7992	8035	3899
Content	916 residues, 4 Ca^2+^, 4 GlcNAc, 5 Na^+^, 1131 water, 3 other	921 residues, 4 Ca^2+^, 8 GlcNAc, 4 Na^+^, 1133 water, 4 other	449 residues, 2 Ca^2+^, 4 Mg^2+^, 9 glycerol, 211 water, 4 other
r.m.s.d. bond lenghts (Å)	0.011	0.012	0.004
r.m.s.d.bond angles (°)	1.57	1.63	0.61
*B*-value (Å^2^): all, protein, ligand, ions, water, other	17.3, 15.6, 10.1, 9.2, 27.3, 30.0	15.8, 14.2, 20.2, 7.5, 24.0, 31.5	45.5, 44.6, 75.5, 62.1, 55.1, 83.8


### Molecular Dynamics Simulations

Molecular dynamics (MD) simulations used the Amber 17 software (Case et al., 2017) with its ff14SB force field for proteins and glycam06_j for glycans. For system setup, Cea1A was protonated by the H++ server (Anandakrishnan et al., 2012) at pH 8 as used before for ITC experiments. The examined glycans, GlcNAc-β1,4-Glc and GlcNAc-α1,4-Glc, were modeled into the binding site according to the Cea1A•*N*,*N*′-diacetylchitobiose complex. For both simulations, three different disaccharide conformers have been used for producing independent trajectories (Supplementary Table S1). The systems were neutralized by xleap at an effective NaCl concentration of 30 mM, and surrounded by a TIP3P water box extending 10 Å around the complex. For initial equilibration under periodic boundary conditions, the six systems were subjected to sequential rounds of minimization and a 100 ps equilibration as NVT ensemble by raising the temperature to 300 K, applying weak restraints to the solute molecules and a Langevin thermostat (random seed, γ = 5 ps^-1^). Finally, constant pressure, restraint-free equilibration to one atmosphere was carried out for 50 ps (Monte-Carlo barostat, pressure relaxation time 2 ps), before each trajectory was allowed to run for 20 ns as NpT ensemble for allowing final convergence. After equilibration 100 ns production trajectories were generated. Overall, we obtained a total of 600 ns overall simulation time from 6 trajectories á 100 ns for analysis. One snapshot was extracted every 0.1 ns, resulting in 1000 snapshots per trajectory, which were combined into 3000 snapshots per studied glycan complex. cpptraj (Case et al., 2017) and dbscan clustering were used for time-dependent dihedral and hydrogen bond analysis (4 minimum points per cluster, and 𝜀 = 1.1, only clusters representing at least 1% of the overall ensembles were accepted). QTiplot was employed to plot results.

### Isothermal Titration Calorimetry (ITC) of Cea1A Domain

Isothermal titration calorimetry measurements were performed with the ITC_200_-System (MicroCal) using a 200 μl cell. A 450 μM solution of Cea1A in SEC-buffer was used as sample. The ligands GlcNAc and *N,N*′-diacetyl-chitobiose were dissolved at 5 mM in SEC-buffer. ITC experiments were performed at 10°C with the first injections being 0.4 μl and 29 injections each with 2 μl. Every injection was applied over a period of 4 s and individual injections were separated by breaks of 360 s. To subtract dilution heat of the carbohydrate, a measurement with the same protocol was performed using only SEC-buffer in the sample cell. Analysis was done using the ITC-data plugin (MicroCal) for Origin 7.0 (Origin-Lab) with the one-binding-site model. Three individual measurements were made per ligand.

### Yeast *in vivo* Adhesion Assay

Adhesion of Cea1A presenting *S. cerevisiae* cells to chitin beads was investigated using the non-adhesive *S. cerevisiae* strain RH2520 (Grundmann et al., 2001) carrying plasmids BHUM2297 (*CEA1A* domain on *ScFLO11BC*) or BHUM1964 (*ScFLO11BC* without A domain as a control). The *CEA1* A domain was isolated by PCR using the primer pairs 5′-AAAAAACCGCGGGATGACAGTGGAAATGGCG/5′-AAAAAAGAGCTCTTCATGGCAGGAGTTCTCATC for Cea1A and chromosomal DNA from *K. pastoris* strain DSMZ 70382 as a template Plasmid BHUM2297 was constructed by integration of the *CEA1A* fragment into plasmid BHUM1964 (Maestre-Reyna et al., 2012) using the restriction sites *Sac*II and *Sac*I.

To monitor binding to chitin beads, plasmid-carrying yeast cells were stained by addition of 50 μl 3,3′-dihexyloxacarbocyanine iodide (DiOC_6_, Santa Cruz Biotechnology, 0.3 mg/ml in 1:9 PBS/EtOH) to 10 OD of exponentially growing cells in 1 ml culture. Cells were mixed and washed with 200 μl of CaCl_2_-supplemented (10 mM) SC-4 medium before resuspension in the same medium. The cell suspension was mixed in a 3:1 ratio with chitin beads (New England Biolabs), which were treated with chitinase (mixture of endo- and exochitinase from *Trichoderma viride*, Sigma-Aldrich) to produce non-reducing terminal GlcNAc ends. 1 ml of SC-4 (+10 mM CaCl_2_) was added to 200 μl of this mixture, and the suspension was gently mixed by inversion followed by 2 min of incubation. The suspension was again mixed by inversion and shortly incubated for 10 s before the supernatant with unbound cells was removed. This washing step was repeated once. Microscopy was performed on beds of 1% agarose with a Zeiss Axiovert 200 M microscope. The cells were examined using differential interference microscopy (DIC) and a GFP filter set for detection of the DIOC_6_-fluorescence (AHF Analysentechnik AG). Cells were photographed with a Hamamatsu Orca ER digital camera (Hamamatsu) and pictures were processed and analyzed using the Volocity software (Perkin Elmer). The amount of free or adherent cells was quantified using 10 comparable image sections of each strain and the program ImageJ (Schneider et al., 2012).

### Immunofluorescence Microscopy

Exposure of the Cea1A domain on the *S. cerevisiae* cell surface was analyzed by immunofluorescence microscopy. For this purpose, cultures of plasmid-carrying strains were grown in low fluorescence yeast medium (Sheff and Thorn, 2004) to an optical density of 1 at 595 nm, before cells were washed three times in PBS/1% BSA. Then, cells were incubated with a monoclonal mouse anti-HA antibody (H3663, Sigma-Aldrich) at a dilution of 1:1000 in PBS/1% BSA for 30 min at 20°C. After three wash steps, cells were incubated in darkness with a Cy3-conjugated secondary goat anti-mouse antibody (C2181, Sigma-Aldrich) at a dilution of 1:10000 in PBS/1% BSA for 20 min at 20°C. After three further washing steps, a Zeiss Axiovert 200 M microscope was used to (i) visualize *S. cerevisiae* cells using DIC and (ii) detect the Cea1A domain at the cell surface using a rhodamine filter set (AHF Analysentechnik AG). Cells were photographed with a Hamamatsu Orca ER digital camera (Hamamatsu) and pictures were processed and analyzed using the Volocity software (Perkin Elmer).

### Data Deposition

The coordinates and structure factors have been deposited at the Protein Data Bank (PDB), Research Collaboratory for Structural Bioinformatics (RCSB), with accession codes 5A3L for Cea1A•GlcNAc, 5A3M for Cea1A•*N*,*N*′-diacetylchitobiose and 6HOS for *Kp*Flo2A•glycerol, respectively. The numbering scheme for Cea1A used in the following refers to Uniprot entry A0A1B2J5V1 and is hence offset by +55 relative to PDB entries 5A3L and 5A3M. The shown glycan V5.1 profiles are deposited at the Consortium for Functional Glycomics (CFG) with the assigned identifier cfg_rRequest #2555 and consistent with earlier profiles from less complete CFG array V4.1 chips (cfg_rRequest #2079).

## Results

### PA14-Domain Containing GPI-CWP Adhesins of *K. pastoris* as a New Subgroup

To search for new, uncharacterized PA14 domain containing adhesins, we used genome mining on the revised *K. pastoris* and *K. phaffii* genome sequences (Love et al., 2016). By using the sequences of *Sc*Flo5A and Epa1A as initial search templates, we identified by PSIBLAST (Altschul et al., 1997) nine orthologs in *Komagataella pastoris* DSMZ70382 (Figure 1A), which belong to PA14 (Pfam-ID: PF07691) or PA14_2 (=GLEYA domain; Pfam-ID: PF10528) domain-containing GPI-CWP adhesins. Although PA14 and GLEYA domains share the same protein topology, GLEYA domains have been only assigned to fungi, mostly as part of cell-surface exposed adhesins like Epa1 and Epa6 from *C. glabrata* (Ielasi et al., 2012; Maestre-Reyna et al., 2012; Diderrich et al., 2015). In contrast, the distinct Pfam family of PA14 domains covers not only other adhesins, but also glycosidases, toxins and other protein topologies. Three orthologs, *Kp*Flo1-*Kp*Flo3, were predicted to harbor an N-terminal PA14-domain with a Ca^2+^-dependent glycan-binding site due to the presence of a so-called D*cis*D motif. The other six, *Kp*Flo4a-*Kp*Flo4f, are apparently devoid of C-type lectin function by lacking this motif including an asparagine essential for Ca^2+^-binding and any cysteines required for intramolecular stabilization by disulfide bond formation (Supplementary Figure S1). For comparison, the related *K. phaffii* strain has seven orthologs for *Kp*Flo1-*Kp*Flo3 and *Kp*Flo4a-*Kp*Flo4d. Additionally, *K. pastoris* and *K. phaffii* harbor each two Flo11-type adhesins, a different type of fungal adhesins, which mediate homophilic interactions and hydrophobic cell wall properties by surface-exposure of clusters of aromatic residues (Kraushaar et al., 2015). Interestingly, *K. pastoris* harbors one further gene product with a PA14-domain (Uniprot entry A0A1B2JD92); here the PA14 domain is part of a GH3-type glycoside hydrolase, which removes glycosides from the non-reducing ends of glycans.

For further classification, we subjected the *K. pastoris* A domains to a phylogenetic analysis by including PA14 domain-containing Flos and adhesins as well as A domains of the PA14_2 type from the *Pfam* database. The maximum likelihood phylogenetic tree shows clustering of these GPI-CWP A domains into six different subgroups (Figure 1B). Five of these subgroups harbor GPI-CWPs from *Saccharomycetaceae*. The Flo5-like A domains form a common cluster (green) and are closely related to each other, while the Epa A domain paralogs from *C. glabrata* and related genera are highly diverse (blue) within their separate cluster. In contrast, the Pwp A domains from *C. glabrata* (Gabaldon et al., 2013), whose function is still unresolved, form a rather small cluster (yellow). Another cluster of putative adhesins is formed by *Kluyveromyces* species (light red). The sixth cluster (brown) belongs to putative adhesins, which miss the conserved CC-motif for linking the *N*- and *C*-terminal ends to the PA14 core, but comprise the D*cis*D motif for Ca^2+^-binding unlike *Kp*Flo4a-*Kp*Flo4f. This cluster is found in fission yeasts and filamentous fungi (Linder and Gustafsson, 2008) as well including several plant and animal pathogens.

The *Komagataella* cluster comprises only orthologs from the *Komagataella* species *K. pastoris* and *K. phaffii*. Our analysis shows that the sequence identity between the different clusters is limited (25–28%), whereas the pairwise identities within the Flo, Epa, and *Komagataella* clusters are in the range of 50–77% (Figure 1C). *Kp*Flo1 (Uniprot entry A0A1B2J5V1; 1720 aa, 186.0 kDa) harbors in its B-region 25 agglutinin-like (ALS) repeats between the A domain (D78-V299) and the C-terminal GPI-anchor motif (Figure 1A). Interestingly, the N-terminus of this GPI-CWP is predicted by TMHMM to form a transmembrane helix (I41-A74) instead of acting as a signal sequence as otherwise found in GPI-CWPs. *Kp*Flo2 bears likewise an A domain (Q33-V256) sharing a high pairwise sequence identity of 59% with *Kp*Flo1A. Unlike *Kp*Flo1 and *Kp*Flo3, the stalk-like B-region of *Kp*Flo2 is not made up of any agglutinin-like repeats. Alignments of *Kp*Flo1A, *Kp*Flo2A and the not further characterized *Kp*Flo3A domain with other fungal adhesins show conservation of both features required for Ca^2+^-binding, the D*cis*D motif in CBL1 and an asparagine in CBL2. These *Kp*Flo A domains also have disulfide bridges that have counterparts in both the Flo and Epa subgroups (Supplementary Figure S1).

### *Kp*Flo1A Domain Specifically Binds β-*N*-Acetylglucosamine

**FIGURE 2:**
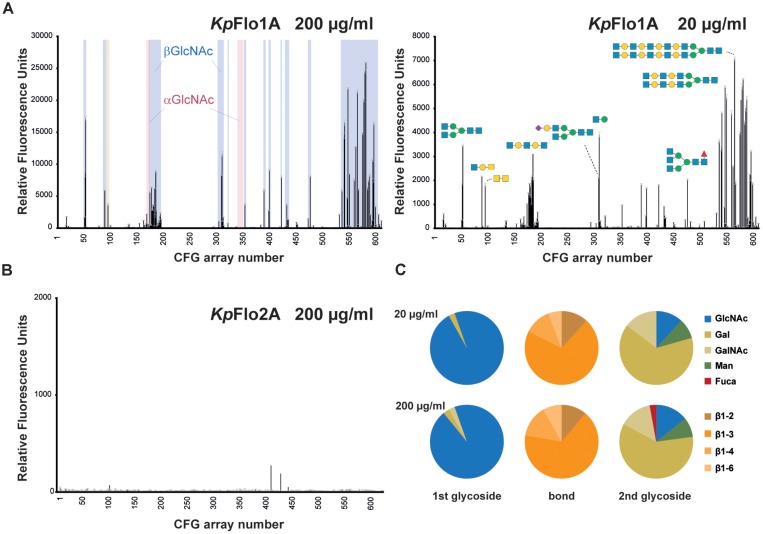
Glycan array screening of *Kp*Flo1 and *Kp*Flo2 A domains. **(A)** Glycan array profiles were done for *Kp*Flo1A at two different protein concentrations (20 and 200 μg/ml). The profiles show distinct peaks in the group of glycans for β-GlcNAc-capped (highlighted in blue, left), but not α-GlcNAc-capped glycans (light red). Only one α-GlcNAc- and a few Gal-capped glycans, where a β-GlcNAc is the second glycoside after the cap show significant signals. Please not that otherwise the group of Gal-capped glycans (glycan 101–173) lacks any signal. Representative glycans are depicted; fully annotated fluorescence datasets are available at the CFG home page (www.functionalglycomics.org; entries: primscreen_5046; primscreen_5047). **(B)** The glycan array profile for *Kp*Flo2A (primscreen_5045) showed no significant hits. **(C)** Pie charts show the glycan types, for which the CFG array binding signals obtained by *Kp*Flo1A exceed 4% of the signal of the best binder and for which terminal disaccharide types are unambiguous. The different glycan types of the terminal (1st glycoside) carbohydrate moiety and the linkage type (bond) to the following (2nd glycoside) carbohydrate unit are color-coded as indicated on the right side.

To examine ligand specificity, we performed large-scale glycan array screening (Blixt et al., 2004) by using fluorophore-labeled *Kp*Flo1A and *Kp*Flo2A domains from *K. pastoris* and the V5.1 glycan chip of the *Consortium for Functional Glycomics*^3^ that carries 611 different glycans. Whereas *Kp*Flo2A lacked any specific glycan binding and represents currently an orphan lectin with unknown target (Figure 2B), we found a high preference of *Kp*Flo1A for terminal *N*-acetylglucosamine (GlcNAc) besides minor contributions from a few glycans bearing terminal galactosyl moieties (Figure 2A). Importantly, most other glycans failed to provide significant fluorescence signals. The preference for GlcNAc capped glycans is further emphasized when comparing only fluorescence signals of >4% of the highest signal (Figure 2C). While this analysis shows that the specificity for the terminal carbohydrate is restricted to terminal β-linked GlcNAc, there is considerable variability of the second carbohydrate and of the connecting glycosidic bond. Accordingly, we named *Kp*Flo1 as chitin-end adhesin 1 (*Cea1*), given its affinity and specificity toward β-GlcNAc capped glycans including chitinous polymers.

### Structures of the Cea1A (=*Kp*Flo1A) and *Kp*Flo2A Domains

**FIGURE 3:**
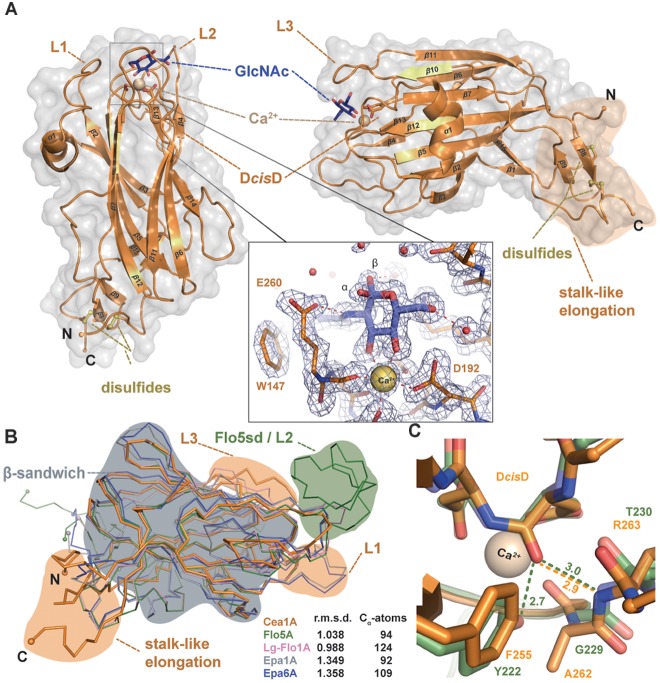
Cea1A has unique structural features distinct from other PA14/Flo5-like adhesins. **(A)** The overall fold of Cea1A shows a β-sandwich, which is derived from the PA14 domain and that is similar to *Sc*Flo5A, Epa1A, and Lg-Flo1A. The Cea1A binding pocket harbors a D*cis*D-motif that coordinates a Ca^2+^ ion (ochre) and confers binding of GlcNAc (blue). Selectivity is achieved by the calcium-binding loop 2 (CBL2) and parts of the flexible loops L1–L3. The *N*- and *C*-terminal parts of Cea1A form a unique stalk-like elongation, which is fixed by two conserved disulfide-bridges (yellow). The inlay shows the GlcNAc-binding site with composite 2mF_o_-*D*F_c_ OMIT electron density as calculated by phenix (light blue; refine mode with contouring level 1.5 σ). **(B)** Superimposition of Cea1A (orange) onto the structures of *Sc*Flo5A (green), Lg-Flo1A (pink), Epa1A (gray), and Epa6A (blue) reveals only minor differences in the PA14-like domain (gray area), but the lack of a *Sc*Flo5A-specific subdomain (green area), a shorter loop L1, a longer loop L3 and a unique stalk-like elongation (orange areas). **(C)** Environment stabilizing the D*cis*D motif in Cea1A (orange) and *Sc*Flo5A (green). In *Sc*Flo5A, the D*cis*D motif is stabilized by a hydrogen bond between its backbone and the hydroxyl group of Y222 (Veelders et al., 2010).

**FIGURE 4:**
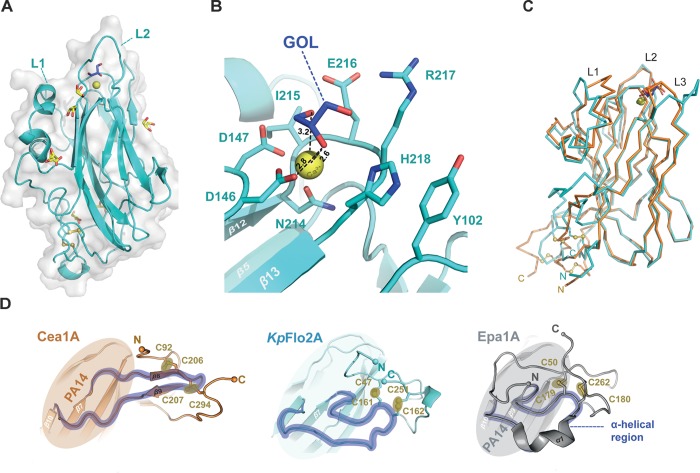
Structure of the orphan lectin *Kp*Flo2A. **(A)** Overall structure of the *Kp*Flo2A domain complexed to glycerol (GOL). **(B)** Putative glycan-binding site with bound glycerol that was derived by long-term soaking in cryobuffer (blue). **(C)** Structural comparison between the Cea1A (orange) and *Kp*Flo2A (cyan) domains. **(D)** The stalk-like subdomain of Cea1A (top, orange) harbors a small β-sheet composed of two strands (β8, β9) that shapes a β-hairpin instead of an irregular, stretched region (*Kp*Flo2A, blue) or a turn/helix-like conformation (Epa1A, gray). In any case, two consecutive and highly conserved disulfide-bonds fuse the *N*-terminal part to the *C*-terminal region via the connecting region.

We were able to crystallize both adhesin domains, Cea1A and *Kp*Flo2A. However, crystallization of the former without any ligand failed to yield well-diffracting crystals. Addition of Ca^2+^ ions with either GlcNAc or the disaccharide N,N’-diacetylchitobiose (GlcNAc-β1,4-GlcNAc) led to orthorhombic Cea1A crystals, which diffracted to 1.7 and 1.8 Å, respectively (Figure 3). The structures of both the Cea1A•GlcNAc and the Cea1A•*N*,*N*′-diacetylchitobiose complexes were solved by molecular replacement (MR) using a search model that was based on the structure of the Epa1A domain (25% sequence identity; Table 1). The orthorhombic crystals comprise four molecules per asymmetric unit. Given that molecules A/B are better defined than C/D, we will refer only to molecule A (Supplementary Figure Supplementary Figure S2A). As expected the 2.1 Å crystal structure of *Kp*Flo2A resembles Cea1A (r.m.s.d. 0.78 Å for 143 Cα atoms) and shows a glycerol molecule coordinated to the Ca^2+^ ion of the glycan binding site (Figure 4). The observed pentagonal-bipyramidal and distorted hexagonal coordination geometries of the Ca^2+^ ions in the *Kp*Flo1A and *Kp*Flo2A complexes, respectively, exclude the binding of Mg^2+^ ions to the glycan binding site, although Mg^2+^ is present in 4:1 and 40:1 excess over Ca^2+^ in the crystallization conditions. The dimeric arrangement of *Kp*Flo2A as found in its crystals is unrelated to Cea1A (Supplementary Figure S2B). These dimers are physiologically irrelevant as formation of a continuous β-sheet structure in *Kp*Flo2A crystals depends on an intervening N-terminal hexahistidine-affinity tag without affecting the putative glycan binding site. Structural differences between Cea1A and *Kp*Flo2A are mainly found for the L1 and L3 loops (for nomenclature refer to (Veelders et al., 2010)) as well as for the neck regions.

As a fungal PA14/Flo5-like adhesin both *Kp*Flo A domains have a conserved β-sandwich, which consists of a six-stranded and a four-stranded β-sheet (Figures 3A, 4A). The binding site for the non-reducing glycan ends is formed by the D*cis*D-motif of CBL1, CBL2 and the loops L1-L3. The latter surround the ligand binding site as previously described for Epa1A. L1 adopts a similar conformation as in the structures of the Lg-Flo1, *Sc*Flo1, and *Sc*Flo5 A domains, whereas L2 lacks the subdomain characteristic of *S. cerevisiae* Flos like *Sc*Flo1 or *Sc*Flo5. Both *Kp*Flo A domains contain the shortest L2 loops of all A domains with known structures (Supplementary Figure S1), leaving thus more space for the binding pocket (Figure 3B). Finally, loop L3 of Cea1A is intimately packed against the GlcNAc ligand by facing directly toward the binding pocket. Unlike other fungal adhesins of the PA14/Flo5-like type, the D*cis*D-motif (D191–D192) lacks a hydrogen bond between a tyrosine from β12 (e.g., *Sc*Flo5A: Y222) and the D*cis*D-backbone. In *Kp*Flo1A and *Kp*Flo2A, this residue is replaced by a phenylalanine (*Kp*Flo1A: F255, *Kp*Flo2A: F212). Consequently, only the remaining hydrogen bond to the backbone A262-R263 (*Kp*Flo2A: A219-V220) stabilizes the *cis*-peptide (Figure 3C).

A unique feature of fungal A domains is the formation of a neck-like, disulfide-bridges comprising subdomain involving their *N*- and *C*-terminal regions (Figure 4D). Although the four cysteine residues of Cea1 and *Kp*Flo2A are conserved, the disulfide bridges formed by these cysteines differ structurally and cause different neck structures. In the case of Epa1A, as well as that of other EpaA and FloA domains, the region of the main chain that is fused to the terminal regions by two disulfide bridges, is flipped directly toward the PA14 domain by forming a short α-helix, which forces the terminal regions into a compact configuration (Figure 4D). In contrast, the comparable region of the main chain of Cea1A forms a regular β-hairpin-like structure (β8–β9) that is fused to the terminal regions and leads to a stalk-like elongation. As a result, the connection between the Cea1A *C*-terminus and the adjacent tandem repeat-rich B region differs structurally from that found in *Kp*Flo2A and other PA14/Flo5-like A domains. Interestingly, for molecules C and D of the Cea1A crystal form, no formation of a neck region and an increased disorder of their N- and C-termini is observed. There, the lack of a disulphide bridge between C207 and C294 coincides with a lack of electron density for the C-terminal stretch D290-A296.

### The Binding Pocket of Cea1A Is Complementary to *N*-Acetylglucosamine

**FIGURE 5:**
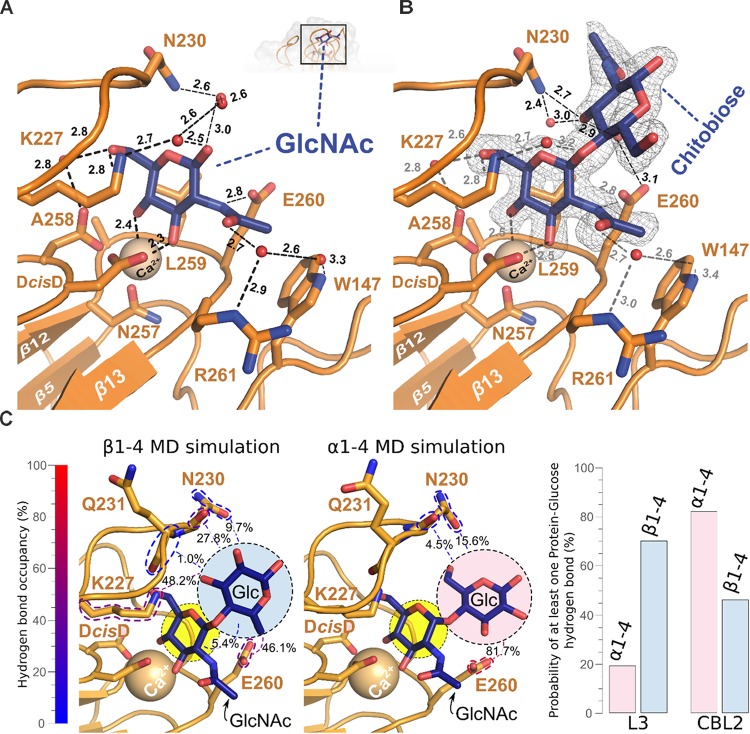
Structural base for the β-GlcNAc specificity of the Cea1A binding pocket. **(A)** The GlcNAc ligand (blue) is recognized by a set of amino acids, the Ca^2+^ ion (golden), which is complexed by the D*cis*D motif in CBL1, and a complex network of water molecules (red). Direct interactions are contributed by E260 in CBL2 and K227 in L3. The water network involves R261 in CBL2, W147 in L2, N230 in L3, D192 in CBL1 and K227 in L3. **(B)** Addition of a second GlcNAc moiety linked via a β1,4-bond (*N*,*N*′-diacetylchitobiose, blue) adds a further hydrogen bond between N230 in L3 and the 3-OH group of the second GlcNAc. This causes a more complex interaction network that is connected to E260 in CBL2 by a further water molecule. The SigmaA-weighted *2mF_obs_-D⋅F_calc_* electron density of N,N’-diacetylchitobiose contoured at 1.2 σ is shown in gray. **(C)** Molecular dynamics simulations and clustering analysis, comparing the binding mode of GlcNAc-β1,4-Glc (β1–4MD) versus GlcNAc-α1,4-Glc (α1–4MD); color scheme as in **(A)**. Each of the structural poses shown here corresponds to the centroid of the highest populated cluster of each simulation, with less populated ones shown in Supplementary Figure S4B. Glucosyl interaction partners are highlighted via colored dashed lines, with the cumulative overall hydrogen bond occupancy shown as a percentage, as well as by the color of the dashed highlights. On the right side, bar diagrams show the cumulative probability of at least one hydrogen bond being formed between L3 and CBL2 at any given time of the made α1–4MD and β1–4MD simulations. The GlcNAc-β1,4-Glc conformation differed from the Cea1A•*N*,*N*′-diacetylchitobiose structure (Supplementary Figure S4A and Supplementary Table S1) as (Klis et al., 2002) the glucose moiety interacts via its 6-hydroxyl with the E260 side-chain, and (Pittet and Conzelmann, 2007) the interaction network with L3 is expanded, e.g., 48.2% of interaction probability for a K227-glucose hydrogen bond. During 27.8% of β1–4 MD simulation, the glucose 2-hydroxyl interacted with the peptide group and not the side-chain of N230 as in the Cea1A•*N*,*N*′-diacetylchitobiose complex (Supplementary Table S2).

The binding pocket of Cea1A shows a complex network of hydrogen bonds and electrostatic interactions and the bound GlcNAc moiety (Figure 5A). As expected, the Ca^2+^-ion is complexed by the D*cis*D-motif and N257 of CBL2. The subsequent residues in CBL2, A258, L259, E260, and R261 (designated as position I–IV), mediate the majority of interactions with the GlcNAc moiety. While A258 and L259 form a hydrophobic part of the binding pocket and interact with C6 of GlcNAc, the rest of the pocket adopt a more polar character. E260 directly interacts with the nitrogen atom of the *N*-acetyl moiety, while R261 contributes, together with W147 from L2, to a hydrogen bond with the acetyl group. Interestingly, in molecule B of the asymmetric unit, R261 adopts an alternative rotamer, where it directly interacts with the *N*-acetyl-group of the ligand. The indole group of W147 forms a hydrophobic patch facilitating van der Waals interactions with the methyl-group of the *N*-acetyl moiety. K227 from L3 above the pocket interacts not only directly with the 6-OH group, but also indirectly via a water-bridged hydrogen bond. Finally, N230, that protrudes from loop L3 acts like a lid above the pocket by contributing to a complex water network above and on the back of the monomeric ligand.

A striking feature of the Cea1A binding pocket are its numerous interactions with the terminal GlcNAc to enclosure it fully. Epa1A also contains a small and deep pocket, that binds to Gal-capped glycans with μM affinity due to a similar number of interactions including a sterically demanding tryptophan from L3 situated above the pocket. In the Flos, *Sc*Flo5A and Lg-Flo1A, the glycan binding site is much wider and shallow than in Epa1A and Cea1A. These differences are reflected by the interaction surfaces between the glycan cap and the different adhesins with values of 192 Å^2^ for Cea1A•GlcNAc, 164 Å^2^ for Epa1A•galactose and only 133 Å^2^ for *Sc*Flo5A•Man.

Interestingly, bound GlcNAc adopts both anomeric forms, i.e., α and β for its C1-hydroxyl group (Figure 3A), where the α-anomeric hydroxyl is even in H-bonding distance to E260 (3.1 Å). In the case of GlcNAc-β1,4-GlcNAc, the second GlcNAc residue contributes only to a few additional interactions with Cea1A (Figure 5B). While the interactions with the first glycan moiety remain unaffected, E260 contributes now to a water-bridged interaction, while N230 in L3 directly interacts with the second GlcNAc moiety. Here, one may note that an α-linked second glycosidic moiety could collide with the sidechains of the CBL2 residues L259 and E260. For understanding the given capacity of Cea1A to discriminate between α- and β-GlcNAc capped glycans (Figure 2A), we performed molecular dynamics simulations (MDS) of Cea1A bound to either GlcNAc-β1,4-Glc or GlcNAc-α1,4-Glc (designated as β1–4 and α1–4 MDS, respectively). To prevent steric interference from the bulky *N*-acetyl moiety of the second GlcNAc moiety, we used instead glucose as second glycoside. Further bias from initially set coordinates was avoided by running three independent 100 ns production dynamics replicas for both systems by employing different start values for the dihedral angles Φ and Ψ, which define the 1,4-glycosidic bond (Supplementary Figure S3 and Supplementary Table S1). In all three β1–4 MDS trajectories the β1,4-linked disaccharide converged to a single conformation (92.0% interaction probability over 3000 analyzed trajectory snapshots). In contrast, the disaccharide conformation in α1–4 MDS was less defined, with Φ–Ψ 2D clustering analysis revealing a total of three major conformations (Figure 5C and Supplementary Figure S3). Furthermore, the simulations did not converge into any of these three conformations, but emerged distinctly based on initial simulation parameters, as evidenced by population distribution analysis of both the glycosidic bond dihedrals and the hydrogen bonding network (Supplementary Figure S4A and Supplementary Tables S1, S2). Only the hydrogen bonding network between E260 of CBL2 and the glucosyl moiety is common to the three conformations (interaction probability: 81.7%). Accordingly, the α1–4 MDS trajectories showed a wide diversity of glucose-Cea1A interactions, none of them appeared to fully stabilize the complex (Supplementary Figure S4B and Supplementary Table S2).

Notably, the solvent accessible surface of Cea1A around the binding pocket is positively charged, although the calculated pI of the whole domain is low (Gasteiger et al., 2005). These positive charges are organized in a patch on the front of the protein (Supplementary Figure S5A, circled), which is a unique feature of Cea1A not found in the structures of *Sc*Flo5A, Epa1A, or Lg-Flo1A. Instead, *Sc*Flo5A possesses a negatively charged surface at the binding pocket, while Epa1A and Lg-Flo1A contain only a small, negatively charged area. We also mapped the conserved amino acid residues onto the surface of the Cea1A structure. This analysis shows a high variability for the PA14-fold with the disulfide bonds and the D*cis*D-motif in CBL1 being the most conserved features (Figure 3C and Supplementary Figures S5B,S5C). The structural rigidity and integrity of the PA14-fold without sequence conservation is a hallmark of many other β-sandwich domains, for example the Ig-type domains (Kraushaar et al., 2015). Interestingly, the major rate of variation in the case of the PA14/Flo5-like adhesins is found in the binding pocket itself, namely in CBL2 and in the flexible loops. This is consistent with the different surface shapes and binding specificities of the binding pocket.

### The Ligand Binding Mode of Cea1A Is Distinct From That of ScFlo5A and Epa1A

**FIGURE 6:**
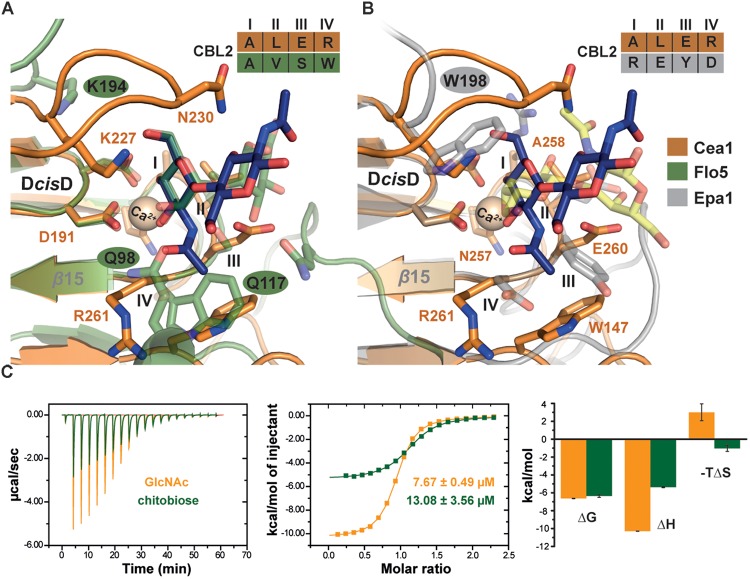
Characteristics of β-GlcNAc binding by Cea1A. **(A)** Comparison of Cea1A•*N*,*N*′-diacetylchitobiose and *Sc*Flo5A•mannobiose complexes. Binding modes of the terminal ligand residue by Cea1A (blue) and *Sc*Flo5A (transparent green) are nearly identical, while orientation and binding modes of the second moiety significantly differ due to different glycosidic bonds (β1,4 in *N*,*N*′-diacetylchitobiose; α1,2 in mannobiose). In Cea1A, K227 lies above the pocket and directly contributes to ligand binding due to the conformation of L3, which shields the binding pocket in a lid-like manner. In *Sc*Flo5A, K194 does not directly bind the ligand due a shorter L3 loop. Residues at CBL2 positions I-IV are indicated. **(B)** Comparison of Cea1A•*N*,*N*′-diacetylchitobiose and Epa1AT-Antigen complexes reveals a different orientation of the ligands, while the steric demand of the two binding pockets is comparable. Remarkably, K227 of Cea1A and W198 of Epa1A have highly congruent positions and are directly involved in ligand binding. **(C)** Isothermal titration calorimetry (ITC) of Cea1A with GlcNAc (orange) and *N*,*N*′-diacetylchitobiose (green). Left, values of the heat released in response to single injections; middle, integration of each peak and fitting to a one-site binding model; right, thermodynamic values of GlcNAc and *N*,*N*′-diacetylchitobiose binding are comparable for ΔG. GlcNAc binding requires a large enthalpic (ΔH) compensation for the negative entropy (ΔS) upon binding, recognition of *N*,*N*′-diacetylchitobiose is entropically more favorable.

Our structural analysis of Cea1A•ligand complexes reveals differences in the ligand binding mode, when compared to *Sc*Flo5A or Epa1A. Given an r.m.s.d. for the primary hexose-ring of 0.3 Å the position of GlcNAc in the Cea1A binding pocket (Figure 6A) matches closely mannoside binding by *Sc*Flo5A. Accordingly, positions I and II of CBL2 are similar, both residues are hydrophobic for packing to C6 of the primary carbohydrate moiety. However, major differences can be found in positions III and IV. At position III, a serine enables *Sc*Flo5A to bind to mannobiose, whereas Cea1A harbors a sterically demanding glutamate, which blocks such an interaction. Furthermore, *Sc*Flo5A stabilizes the disaccharide orientation by residue Q117 of its Flo-specific subdomain. At position IV of CBL2, *Sc*Flo5A harbors a tryptophan, which sterically blocks the binding of GlcNAc together with Q98 of the *Sc*Flo5A subdomain, thereby shielding this part of the pocket. For specific interaction with the acetyl group of GlcNAc, Cea1A instead contains an arginine at position IV.

Comparison with Epa1A shows a different orientation of the terminal hexose moiety (Figure 6B). Compared to the Cea1A-GlcNAc complex, the galactoside is rotated in the inner pocket of Epa1A by almost 180 degrees along the axis of the glycosidic bond. In Epa1A the indole group of W198 from L3 dictates the coplanar orientation of the galactosides and a major factor for high-affinity binding (Maestre-Reyna et al., 2012), whereas Cea1A harbors a lysine residue, K227, at the corresponding position for packing with the pyranose moiety. Finally, W147 in L2 of Cea1A, which forms part of a hydrophobic region that packs to the acetyl group, has no comparable counterpart in the A domain of Epa1A, *Kp*Flo2A (Y102) or other PA14/Flo5-like adhesins. In summary, these data show that while Cea1A shares some of the structural characteristics found in Flos and epithelial adhesins, it confers ligand binding by a clearly distinct mode not observed in other PA14/Flo5-like adhesins so far.

### Specific Recognition of β-GlcNAc-Capped Glycans *in vitro* and *in vivo*

To further investigate whether the specificity for GlcNAc binding by Cea1A is matched by high affinity, we used isothermal titration calorimetry to determine thermodynamic parameters for binding of GlcNAc and GlcNAc-β1,4-GlcNAc (Figure 6C). Both ligands bind enthalpically driven to Cea1A with a smaller ΔH contribution caused by *N*,*N*′-diacetylchitobiose (-5.4 kcal^∗^mol^-1^) than by GlcNAc (-10.3 kcal^∗^mol^-1^). However, *K*_D_ values of 7.6 μM for GlcNAc and 13.1 μM for *N*,*N*′-diacetylchitobiose, respectively, are similar (Table 2) and point to tight interaction, especially when compared to *Sc*Flo5A or Lg-Flo1A. *Sc*Flo5A mediates weak, but specific α-Man capped glycans with mM affinities; Lg-Flo1A recognizes Man-1-phosphate in the higher μM range (Veelders et al., 2010; Maestre-Reyna et al., 2012; Sim et al., 2013). Only Epa1A binds to galactose-capped glycans with μM affinities (Veelders et al., 2010; Maestre-Reyna et al., 2012; Sim et al., 2013). Given similar free enthalpies (ΔG) for GlcNAc and GlcNAc-β1,4-GlcNAc binding to Cea1 (Figure 6C) binding of GlcNAc by Cea1A is entropically disfavored. The reduced entropic loss upon *N*,*N*′-diacetylchitobiose binding may reflect the lack of interactions between the second GlcNAc moiety and the adhesin binding site, the loss of hydrogen bonds between the 1-hydroxyl group and bridging water molecules (Figure 5B) as well as the release of several ordered water molecules from the pocket upon accommodation of the second GlcNAc moiety due to packing interactions between the acetyl group and the side chain of N230. As expected for a C-type lectin binding mode, depletion of Ca^2+^ ions by addition of 5 mM EDTA causes a dramatic loss of ligand binding (Supplementary Figure S6C). Glycan binding by Cea1A is very specific given that glucose, glucosamine or other like *N*-acetylneuraminic acid (Neu5Ac) failed to bind under analogous ITC conditions (Supplementary Figure S6A). Interestingly, for glucose we could observe weak binding by Cea1A with a *K*_D_ of 2.38 mM, which could be further validated by fluorescence titration (Supplementary Figures S6B,D). The loss of three orders of magnitude for binding underlines the role of the *N*-acetyl group of GlcNAc for cognate interactions within the Cea1A binding pocket.

**Table 2:** Thermodynamic parameters of Cea1A ligand recognition.

Thermodynamic parameters	Cea1A•GlcNAc	Cea1A•*N*,*N*′-diacetylchitobiose
*K*_D_ (μM)	7.67 ± 0.49	13.08 ± 3.56
ΔH (kcal^∗^mol^-1^)	-10.29 ± 0.01	-5.37 ± 0.05
-TΔS (kcal^∗^mol^-1^)	3.00 ± 0.94	-1.04 ± 0.33
ΔG (kcal^∗^mol^-1^)	-6.64 ± 0.03	-6.36 ± 0.16
*N* (sites)	0.919 ± 0.001	1.046 ± 0.007


**FIGURE 7:**
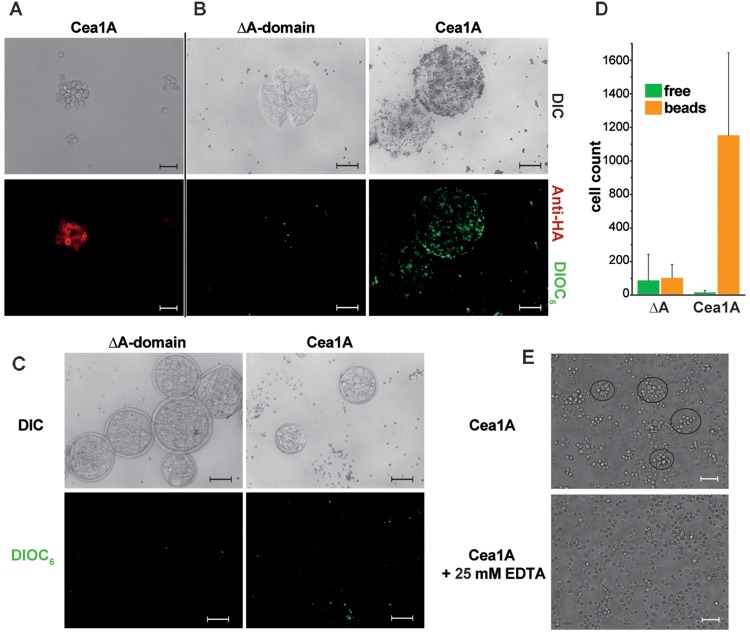
Cea1A presented on *S. cerevisiae* cells confers adhesion to chitin *in vivo*. **(A)** Immunofluorescence-labeling of Cea1A on the surface of the BHUM2297 harboring *S. cerevisiae* strain. The scale bar corresponds to 20 μm. **(B)** Cea1A-presenting and DIOC_6_–labeled *S. cerevisiae* cells (BHUM2297) as well as control cells lacking an A domain were incubated with chitinase-pretreated chitin beads. Cea1A-presenting cells decorate the chitin beads, whereas only few cells of the negative control without A domain are left after washing of the beads. The scale bars correspond to 100 μm. **(C)** Like **(B)** but using untreated chitin beads. No specific adhesion to chitin beads is visible for the control and Cea1A presenting cells. **(D)** Free and bead-adhering cells were counted for both *S. cerevisiae* strains. In the ΔA domain strain no difference between free and adhering cells is found, while in the Cea1A-expressing strain almost all cells adhere to chitin beads if incubated with pretreated beads for exposing free β-GlcNAc caps. **(E)** Surface presentation of Cea1A causes formation of *S. cerevisiae* aggregates comprising about 10 cells, which can be dissolved by addition of 25 mM EDTA. The scale bar corresponds to 20 μm.

To investigate whether Cea1A recognizes chitinous polymers under *in vivo* conditions, we employed a heterologous expression system for A domain presentation on the cell surface of a non-adhesive *S. cerevisiae* strain (Maestre-Reyna et al., 2012). Successful maturation and surface presentation of Cea1A was monitored by immunofluorescence microscopy (Figure 7A). Chitin binding was tested by incubation of control and Cea1A presenting strains with either untreated chitin beads or with chitin beads that were pre-treated by partial digestion with chitinase, which leads to unmasking of non-reducing β-GlcNAc ends (Figures 7B–D). We found that cells of control strains failed to adhere to untreated or pre-treated beads, whereas Cea1A-presenting cells efficiently bound to enzymatically pre-treated beads. Interestingly, adhesion to non-reducing ends of chitinous polymers coincides with the formation of small, microscopic flocs of Cea1A presenting *S. cerevisiae* cells, which can be dissolved by addition of 25 mM EDTA as calcium chelator (Figure 7E). Overall, our data clearly demonstrate the ability of Cea1A to bind to β-GlcNAc capped glycans with high affinity and specificity, e.g., for conferring efficient adhesion to non-crystalline chitin.

## Discussion

Although the methylotrophic yeasts *K. pastoris*, formerly known as *P. pastoris*, and its close relative *K. phaffii* represent the most commonly used yeast species for the production of recombinant proteins (Zahrl et al., 2017), including biopharmaceuticals and industrial enzymes, there is almost no knowledge of their adhesive properties and interactions with environmental surface cues. In contrast, the model organism *S. cerevisiae* has been extensively characterized in terms of the genetic and biochemical base for flocculation and surface adhesion (for overview refer to Verstrepen and Klis, 2006; Dranginis et al., 2007; Bruckner and Mosch, 2012; Lipke, 2018). Recently, surface attachment and pseudohyphae formation of *K. pastoris* cells could be successfully suppressed by deletion of an ortholog of the *FLO8* gene, a gene that was known before to act as master regulator for adhesin genes in *S. cerevisiae* (Rebnegger et al., 2016).

In contrast to the first draft genomes of *Komagataella* species, recently revised genomic and transcriptomic data (Love et al., 2016) allowed the identification of a set of genes coding for 9 (*K. pastoris*) and 7 (*K. phaffii*) PA14/Flo5-like domain containing GPI-CWP adhesins, respectively. Three of them *Kp*Flo1-*Kp*Flo3 are well conserved also in *K. phaffii* given pairwise sequence identities of 89, 86, and 94% for their A domains. In this study, we characterized the A domains of two of them. *Kp*Flo2 represents currently an orphan lectin, as we found no target glycan when screening CFG glycan arrays, which are biased toward mammalian glycans by omitting microbial and plantal glycan structures. In the case of the related Epa A domains from *C. glabrata* 5 of 17 analyzed A domains likewise lacked any specificity (Diderrich et al., 2015). Apparently, the repeated occurrence of fungal lectins/adhesins with yet unknown specificity may be rather common given the structural diversity of glycans outside the mammalian world.

However, our study uncovers Cea1 as the first known fungal cell wall adhesin that strictly recognizes β-GlcNAc caps of chitinous polymers and other glycans with μM affinity and is able to mediate specific cell adhesion, e.g., to non-crystalline chitin. Soluble lectins specific for GlcNAc moieties have been so far discovered in basidiomycetes (Jiang et al., 2012; Audfray et al., 2015; Ribeiro et al., 2017). Although these lectins may have diagnostic potential, they suffer from low affinity in the high μM range, their dependence on avidity for efficient binding and the lack of discrimination between β- and α-linked GlcNAc moieties. At first sight the preference for β-GlcNAc capped glycans resembles the preference for β-galactosyl caps by Epa1 from *C. glabrata* as steric discrimination is driven in the latter by bulky residues at positions II and III of CBL2 as well (Cea1: L259, E260; Epa1: E227, and Y228). Indeed, our MD simulations show that Cea1 position III is often contacted by the reducing ends of both β- and α-linked disaccharides. While the interaction with the β-linked glucose moiety is strong (46.1% interaction probability), the α-glycosidic bond initially appears to produce an even more interaction (82.1%, Figure 5C, right panel). However, closer inspection shows that in MD simulations with β1,4-linked GlcNAc only the 6-hydroxyl of the second glycosidic moiety is responsible for glycan-E260 interactions, whereas α1,4-linked GlcNAc exhibits heterogeneous interactions between its second glycosidic group and position III. This indicates that interactions between the second glycosidic group and position III are more transient than for β1,4-linked GlcNAc (Supplementary Table S2). Our MD simulations further suggest that both N230 and K227 of L3 interact tightly with the second glycosidic group (Figure 5C and Supplementary Figure S4B), if it is β-linked to the GlcNAc cap (β1–4 MDS: 70.0% interaction probability; α1–4 MDS: 19.2%; Supplementary Table S2). Accordingly, we propose that, unlike in Epa1A, the L3 loop of Cea1Â actively determines the preference for β-GlcNAc caps.

Our data show that Cea1A harbors features distinct from other characterized PA14/Flo5-like adhesins. One feature of Cea1A not found in other PA14/Flo5-like adhesins is the neck-like subdomain that fixes the *N*- and *C*- termini to the core of the A domain and forms an elongated stalk for the linkage with the B region. The B region of GPI-CWPs is predicted by *de novo* modeling of *Candida albicans* Als-adhesins to consist of small β-sheets and has been shown to be functionally important in *S. cerevisiae* for the presentation of A domains on the cell wall surface. In Cea1, the neck-like subdomain projects the repetitive B region into a direction of nearly 45° relative to the A domain (Figure 4D) (Verstrepen et al., 2005; Frank et al., 2010). A second feature is the unusually narrow binding site of Cea1A for the terminal glycan moiety due to direct interaction of K227 from the flexible loop L3 with GlcNAc. This enables a high number of interactions between Cea1A and the terminal GlcNAc moiety, a feature that is also reflected by the particular charge distribution around the binding pocket. Although a lysine residue that corresponds to K227 of Cea1A is also present in L3 of *Sc*Flo5A, the rest of this loop completely differs from Cea1A and adopts a different conformation (Figure 3B). Accordingly, Cea1A and *Sc*Flo5A exhibit clearly distinct ligand binding modes, even though the orientation of the primary carbohydrate is similar. The small pocket and binding mode of Cea1A cause restricted ligand specificity for terminal GlcNAc, whose high affinity is sufficient to confer binding to non-crystalline chitin *in vivo*.

Can we delineate a biological function from the characteristics of Cea1A to recognize terminal β-GlcNAc caps *in vivo*? An interesting observation of our study is the formation of small flocs by *S. cerevisiae* strains, which present Cea1A on their cell surfaces (Figure 7E). Here, Cea1A might be able to confer a type of flocculation by binding to exposed, non-crystalline chitin of the fungal cell wall. In *S. cerevisiae* and other yeasts, the total amount of chitin corresponds to only 1–2% of the dry weight of unstressed cells (Orlean, 2012) and is mostly deeply buried in the cell wall under a thick layer of mannoproteins and β-glucans, with only minor amounts being exposed at the cell surface, e.g., in bud scars. This might explain, why flocs induced by Cea1A and sparse surface chitin are considerably smaller than flocs formed by *Sc*Flo5A/α-mannoside interactions. Interestingly, *Kp*Flo1 does not belong to the GPI-CWPs, because it is anchored to the plasma membrane by an N-terminal transmembrane helix. An alternative function may be hence a stabilization of the fungal cell wall by Cea1, because its location would foster an interaction with the inner chitin layer of the fungal cell wall. However, such a cell-wall stabilizing function is likely restricted to *K. pastoris*, because the Flo1 ortholog of *K. phaffii* corresponds to a conventional GPI-CWP, which are commonly exposed on the outer cell wall.

The large set of putative PA14/Flo5-like adhesins present in *K. pastoris* (Figure 1A) and *K. phaffii* indicate that these proteins might confer adhesion in processes other than flocculation, such as the efficient binding to solid substrate surfaces or host cells. It is interesting to note, that decomposing wood has been suggested to represent the natural habitat of yeasts of the genus *Komagataella* (Phaff and Knapp, 1956; Kurtzman, 2011). Furthermore, a number of yeast species are associated with insects, including wood-feeding ones, where they play a role as symbionts or pathogens (Suh et al., 2005; Prillinger and König, 2006; Gibson and Hunter, 2010). In the case of *S. cerevisiae*, social wasps have for example been found to act as environmental vectors (Stefanini et al., 2012). Yeasts of the related *Pichia* clade were indeed found in the guts of the passalid beetle *Odontotaenius disjunctus* and in specialized organelles used for myco-symbionts, where they support the digestion of complex carbohydrates (Suh et al., 2003; Suh and Blackwell, 2004). *K. pastoris* was initially found to be closely associated with the fruit fly *Drosophila melanogaster* (Shihata and Mrak, 1952; Kurtzman, 2011). Given the fact that these insects not only possess chitin-based exoskeletons, but also use chitinous polymers to build up their digestive tracts, Cea1A and other members of the *Komagataella* subfamily of PA14/Flo5-like adhesins might be crucial for different yeast-insect interactions. This hypothesis is further supported by the fact that ascomycetes possess not only free but also secreted chitinases, which are capable to generate free β-GlcNAc ends from polymeric or crystalline chitin on host surfaces (Hartl et al., 2012).

In summary, our discovery of a novel subgroup of PA14/Flo5-like adhesins from *Komagataella* provides insights into the structural and functional complexity and evolution of fungal adhesins and their ability to specifically recognize a wide variety of carbohydrates. In contrast to the Epa family, whose broad phylogenetic relationships indicate high variability, the cluster of PA14/Flo5-like adhesins from *Komagataella* is relatively small, and in this regard comparable to the Flos (Figure 1B). Furthermore, the phylogenetic distances between the different subgroups of fungal adhesins appear to be comparable. The latter provokes the hypothesis that the functional diversity of PA14/Flo5-like adhesins has evolved from a common ancestor by variation of a conserved structural motif and was driven by adaptation of different yeast species to new habitats and ecological niches. For example, the genomes of pathogenic *Candida* species have a significantly higher number of genes for Epa-like adhesins than non-pathogenic strains (Gabaldon et al., 2013). Given their number of *Kp*Flo genes *Komagataella* species apparently follow this trend for functional specialization of adhesins (Figure 1B).

## Author Contributions

L-OE, MK, and MV designed the research. MK, SB, JS, and NW performed the research. MK, SB, and L-OE analyzed the data. MK and SB contributed to *in vivo* experiments. MM-R performed the molecular dynamics analyses. L-OE, H-UM, MM-R, and MK wrote the manuscript. All authors reviewed the results and approved the final version of the manuscript.

## Conflict of Interest Statement

The authors declare that the research was conducted in the absence of any commercial or financial relationships that could be construed as a potential conflict of interest.

## Footnotes

1 http://tree.bio.ed.ac.uk/software/figtree

2 www.functionalglycomics.org

3 www.functionalglycomics.org

## Abbreviations

A domain: adhesion domain
CBL: calcium binding loop
Cea: chitin end adhesin
CWP: cell wall-associated protein
Epa: epithelial adhesin
Flo: flocculin
Gal: galactose
GlcNAc: *N*-acetyl-D-glucosamine
GPI: glycosylphosphatidylinositol
*K*_D_: dissociation constant
Man: mannose
