# Supplementary material for: Structural and Functional Characterization of PA14/Flo5-Like Adhesins From Komagataella pastoris
Source: Front Microbiol. 2018 Oct 30;9:2581. doi: 10.3389/fmicb.2018.02581 (PMC6218569; doi:10.3389/fmicb.2018.02581)
Supplement: Supplementary file 1 [file Data_Sheet_1.docx]

**SUPPLEMENTARY Figure 1**


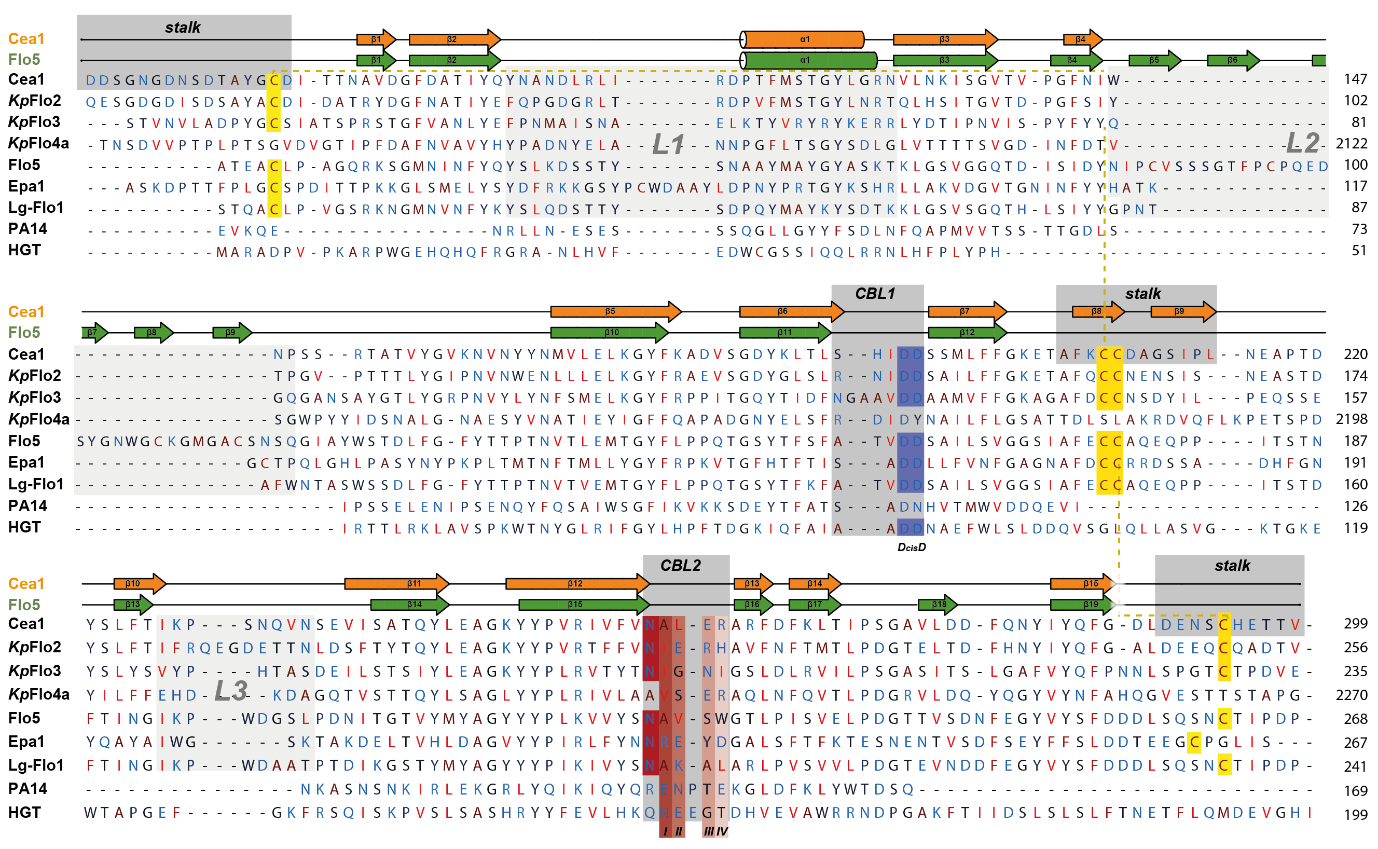


FIGURE S1. **Alignment of *Kp*Flo A domain sequences with other PA14/Flo5-like domains.** An alignment of *Kp*Flo1A-*Kp*Flo3A as well as of *Kp*Flo4aA and representatives of the PA14/Flo5-like family (Uniprot-entries: *Sc*Flo5, P38894; Epa1 adhesin from *C. glabrata*, Q6VBJ0; LgFlo1, B3IUA8; PA-14, P13423; human galactosaminyl transferase HGT, Q8N9V0­­) was generated using the T-Coffee-algorithm (20) and combined with the structural annotation of the secondary structure of Cea1A and *Sc*Flo5A. Regions forming the stalk, CBL1 or CBL2 are highlighted in grey. The stalk region is formed mainly by a small *β*-sheet (*β*8, *β*9) and the *N*- and *C*-terminus of the A domain. Positions I-IV of CBL2, that are central constituents of the binding pocket, are indicated. The highly conserved D*cis*D motif is marked in blue and the conserved disulfide-bonds in yellow.

**SUPPLEMENTARY Figure 2**

**
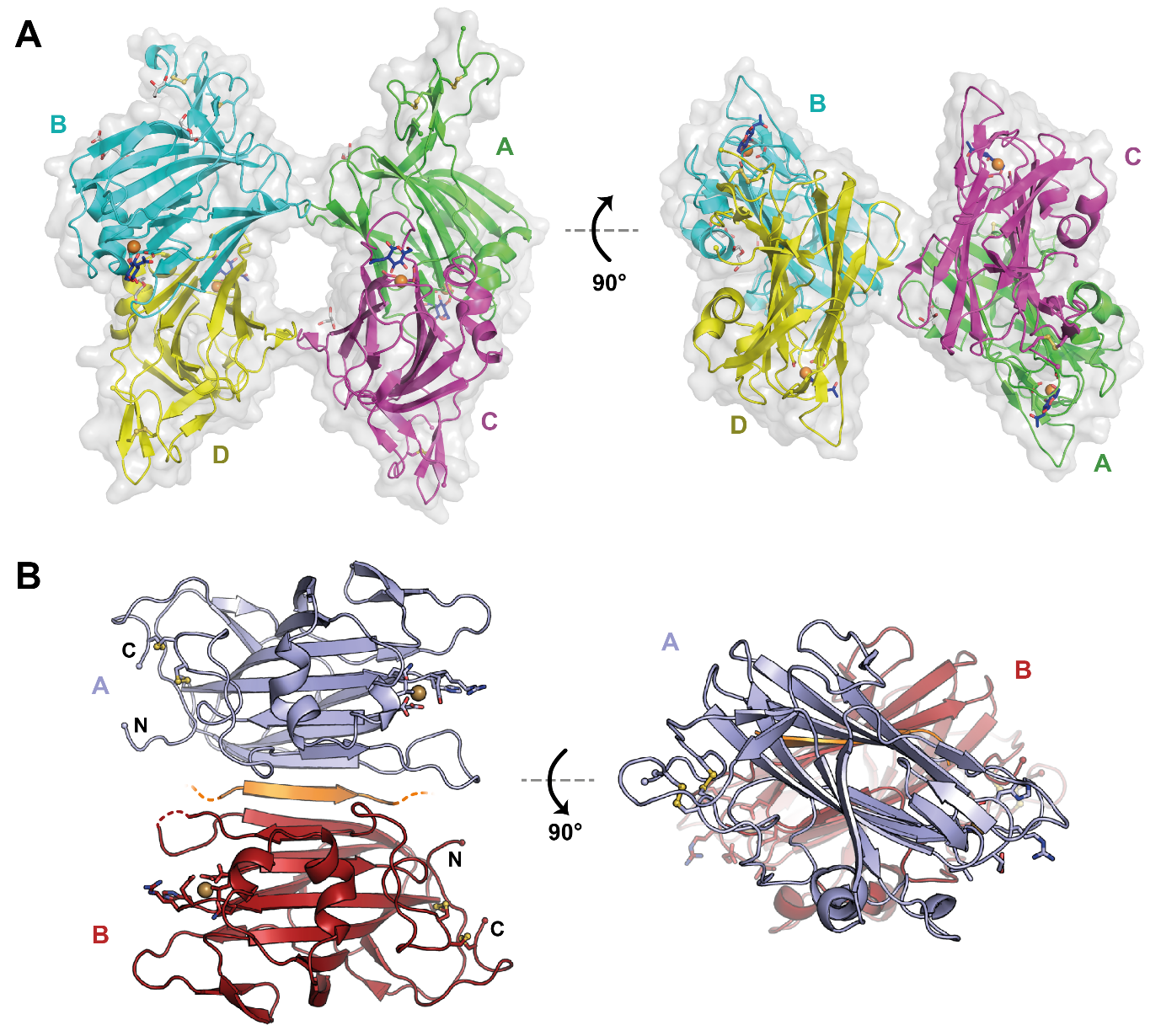
**

FIGURE S2. **Crystal packing of *Kp*Flo A domains.** *A*. The four molecules (A, B, C, D) of the orthorhombic crystal form of Cea1A are arranged in an antiparallel manner and form with their β-sandwiches a continuous 10-pleaded β-sheet by edge-to-edge packing. The chains are shown with Ca^2+^ (orange) and bound GlcNAc ligands (blue). Molecules A-D are almost identical with a maximum r.m.s.d. of 0.22 Å between two Cea1A molecules. Due to crystal packing, two Cea1A molecules show pairwise a higher structural similarity to each other. *B*. Dimer of the KpFlo2A domain as present in the asymmetric symmetry unit. The molecules A (blue) and B (red) form a continuous β-sheet via parts of an His_6_-affinity tag (orange).

**SUPPLEMENTARY fIGURE 3**


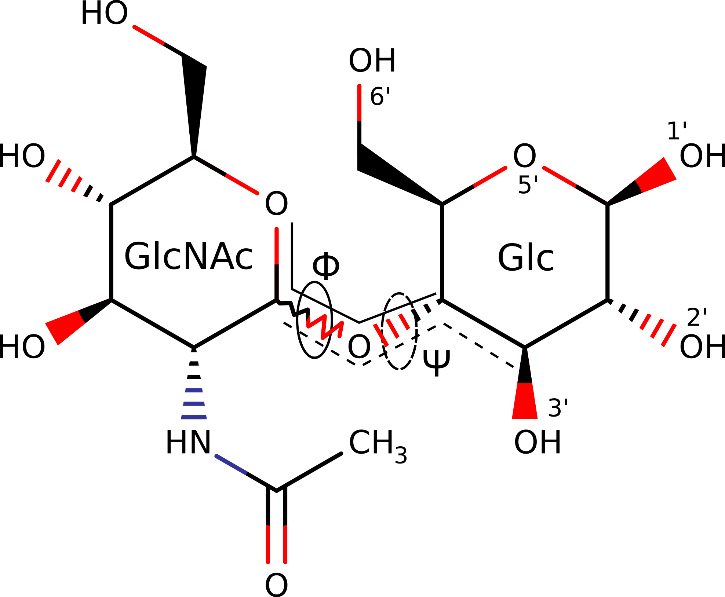


Figure S3 Definition of the Φ-Ψ angles, as well as of the glucosyl positions for all simulations.

**supplementary figure 4**


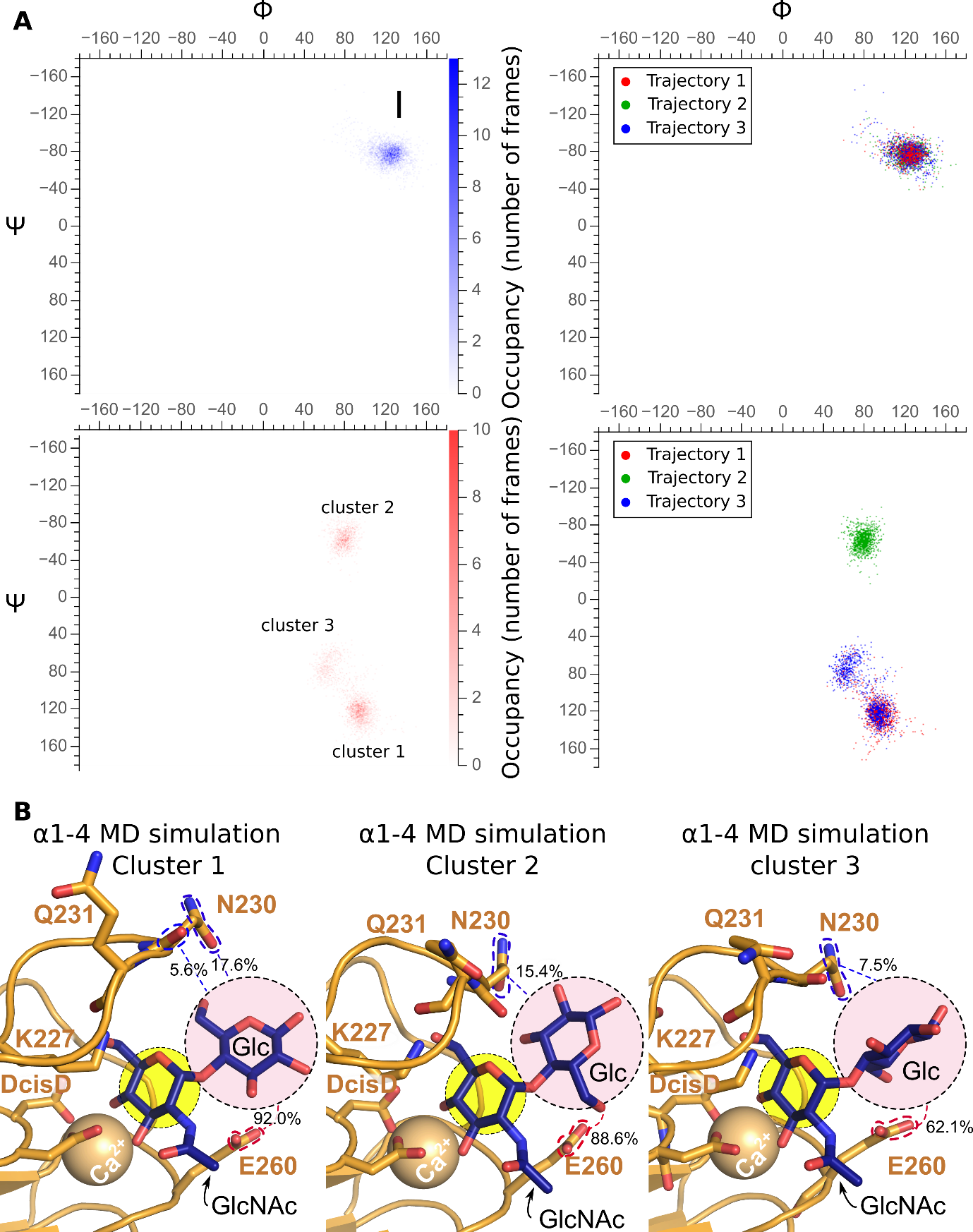


Figure S4 **Clustering analysis of α1-4 and β1-4 MDS**. A. 2D Φ-Ψ plots for β1-4 MDS (top) and α1-4 MDS (bottom) showing the population density for each cluster (left) or the value distribution by trajectory replica (right). Note that β1-4 MDS presents a single, clearly defined cluster with highly overlapping values throughout the overall simulation, on which all three trajectory replicas converge (Table S1). On the other hand, α1-4 MDS shows a total of three clusters, resulting in a high degree of discrepancy between the overall Φ and Ψ values for the entire simulation and those of the individual clusters (Tables S1,S2). Furthermore, trajectory replicas do not converge, as clusters 1 and 3 are populated exclusively by trajectories 1 and 3, while cluster 2 only by trajectory 2. B. detailed, per-cluster description of the glucosyl hydrogen bonding network with nearby interaction partners in α1-4 MDS. Here, the glycan ligand (blue) is bound by Cea1A and Ca^2+^ (golden). Waters have been stripped for clarity. Glucosyl interaction partners are highlighted via colored dashed lines, with the cumulative overall hydrogen bond occupancy shown as a percentage, as well as by the color of the dashed highlights.

**SUPPLEMENTARY Figure 5**

**
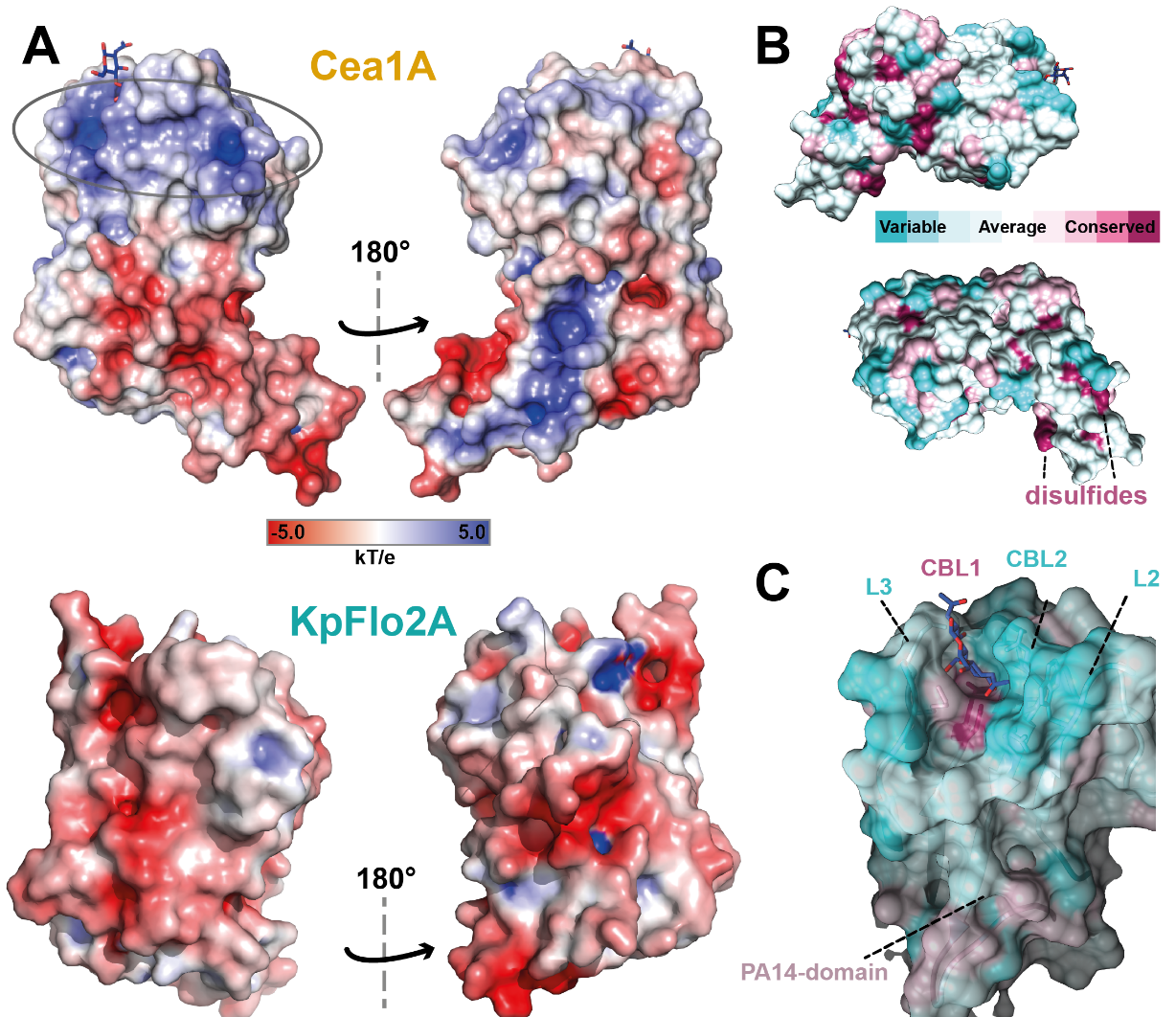
**

FIGURE S5. **Surface properties of *Kp*Flo A domains.** *A*. The electrostatic surface of Cea1A (top) and KpFlo2A (bottom) was generated by APBS at 0.15 M NaCl concentration (20). In Cea1A, a patch of positively charged residues (blue) enclose the binding pocket (grey circle), whereas KpFlo2A carries a negatively charged surface near the binding pocket like other fungal PA14/Flo5-like A domains. *B*. Conservation of surface properties of Cea1A. Structural models of Cea1A and the binding pocket (*C*.) depict conserved and variable surface residues according to ConSurf analysis. The highest conservation is found for the D*cis*D-motif and the two disulfide bridges.

**SUPPLEMENTARY Figure 6**

**
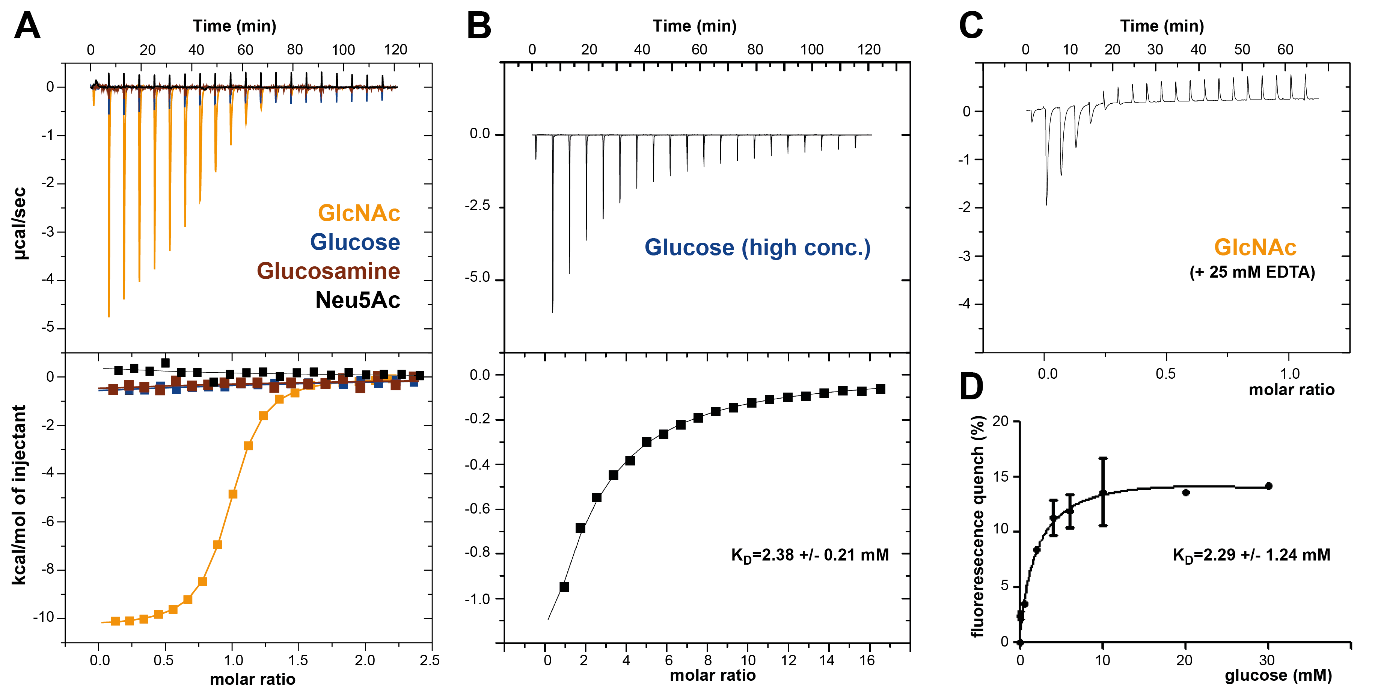
**

FIGURE S6. **Binding of GlcNAc by Cea1A is specific and depends on Ca^2+^.** *A*. Unlike GlcNAc, glucose, glucosamine and *N*-acetylneuraminic acid (Neu5Ac) fail to bind to Cea1A under ITC conditions when using stock concentrations of 5 mM. The upper part shows the values of the heats released in response to single injections, while integration of each peak and corresponding fits to a one-site binding model are depicted as curves in the diagram below. *B*. Titration of 500 µM Cea1A/Δ291-299 using a 40 mM stock of glucose for injections. *C*. Titration of 450 µM Cea1A using 5 mM EDTA as added stock effectively ablates binding to GlcNAc in isothermal titration calorimetry (ITC) analysis. Small peaks visible for the first three injections may indicate that Ca^2+^ removal from the binding pocket is incomplete. D. Fluorescence titration of 11.5 µM Cea1A/Δ291-299 with glucose using λ_exc_=280 nm and λ_em_=295 nm for recording of triplicates.
