# Supplementary material for: Structural and Functional Characterization of PA14/Flo5-Like Adhesins From Komagataella pastoris
Source: Front Microbiol. 2018 Oct 30;9:2581. doi: 10.3389/fmicb.2018.02581 (PMC6218569; doi:10.3389/fmicb.2018.02581)
Supplement: Supplementary file 2 [file Table_1.DOCX]

**Table S1 Average dihedral angles defining the glycosidic bond during *α*1-4 MDS and *β*1-4 MDS.** In the main text, values are given per cluster, while here, per trajectory. For trajectory 1, the same Ф/Ψ conformer as that of the N,N’-diacetylchitobiose template was chosen; for trajectory 2, Ф/Ψ torsion angles were derived from xleap (34) energy minimized GlcNAc-α1,4-Glc. Finally, for trajectory 3, Ф/Ψ values were suitably adapted to place the glucose moiety of GlcNAc-α1,4-Glc to a similar position as the second GlcNAc moiety in the Cea1A•N,N’-diacetylchitobiose structure.

|  | Initial Values | | Average | |
| --- | --- | --- | --- | --- |
| *α*1-4 MDS | | | | |
|  | Φ | Ψ | Φ | Ψ |
| Overall | N/A | N/A | 83.5±13.7 | 50.5±83.8 |
| Trajectory 1 | -114.0 | 70.7 | 80.9±16.6 | 99.1±26.0 |
| Trajectory 2 | 69.4 | 97.5 | 91.5±11.9 | 116.9±17.3 |
| Trajectory 3 | 78.5 | -52.6 | 78.1±6.9 | -64.4±9.7 |
| *β*1-4 MDS | | | | |
|  | Φ | Ψ | Φ | Ψ |
| Overall | N/A | N/A | 122.5±11.3 | -78.4±9.2 |
| Trajectory 1 | -114.0 | 70.7 | 121.8±11.4 | -78.9±8.9 |
| Trajectory 2 | 69.4 | 97.5 | 122.4±12.1 | -78.5±10.1 |
| Trajectory 3 | 78.5 | -52.6 | 123.4±10.4 | -77.8±8.5 |
