# Supplementary material for: Structural and Functional Characterization of PA14/Flo5-Like Adhesins From Komagataella pastoris
Source: Front Microbiol. 2018 Oct 30;9:2581. doi: 10.3389/fmicb.2018.02581 (PMC6218569; doi:10.3389/fmicb.2018.02581)
Supplement: Supplementary file 3 [file Table_2.DOCX]

**Table S2 Occupancy of the H-bonding networks formed by second glycosidic moiety in *α*1-4 MDS and *β*1-4 MDS.** Compare to Fig. S2 for details. Only hydrogen bonds with either occupancy in cluster, or overall occupancy, equal to or greater than 1% are listed here.

| *α*1-4 MDS | | | | |
| --- | --- | --- | --- | --- |
| Cluster | **Acceptor** | **Donor** | **Occupancy in**  **cluster (%)*** | **Overall occupancy (%)^†^** |
| 1 | N230 δO | glucosyl 6-OH | 16.9 | 9.7 |
| 1 | glucosyl 6’O | N230 peptide NH | 8.4 | 4.2 |
| 1 | E260 εO | glucosyl 3-OH | 92.0 | 43.8 |
| 2 | N230 δO | glucosyl 2-OH | 6.3 | 2.4 |
| 2 | N230 δO | glucosyl 3-OH | 4.0 | 1.5 |
| 2 | glucosyl 2-OH | N230 δNH | 4.4 | 1.7 |
| 2 | E260 εO | glucosyl 6-OH | 88.6 | 37.9 |
| 3 | N230 δO | glucosyl 6-OH | 6.5 | 0.3 |
| 3 | E260 εO | glucosyl 6-OH | 62.1 | 37.9 |
| *β*1-4 MDS | | | | |
| Cluster | **Acceptor** | **Donor** | **Occupancy in**  **cluster (%)*** | **Overall occupancy (%)^†^** |
| 1 | glucosyl 3-OH | K227 ζNH | 48.6 | 48.2 |
| 1 | N230 peptide O | glucosyl 2-OH | 27.7 | 27.0 |
| 1 | N230 δO | glucosyl 2-OH | 7.1 | 7.2 |
| 1 | N230 δO | glucosyl 3-OH | N/A | 1.2 |
| 1 | N230 δN | glucosyl 2-OH | 1.1 | 1.1 |
| 1 | E260 εO | glucosyl 6-OH | 47.3 | 46.1 |

^*^Occupancy in cluster corresponds to the number of frames where the interaction was present divided by the number of frames in the corresponding cluster.

^†^Overall occupancy corresponds to the number of frames where the interaction was present divided by the 3000 snapshots defining the entire MDS in either case.
